# Effect of Block Copolymer Architecture on Lead(II) Bromide (PbBr_2_) Dissociation and Complexation via Surface Adsorption in a Selective Solvent

**DOI:** 10.1021/acs.langmuir.6c02844

**Published:** 2026-08-27

**Authors:** Ya-Sen Sun, Yu-Chieh Chen

**Affiliations:** Department of Chemical Engineering, 34912 National Cheng Kung University, Tainan 701, Taiwan

## Abstract

In this study, we systematically investigate how the molecular weight and block volume fraction of polystyrene-*block*-poly­(ethylene oxide) (PS-*b*-PEO) diblock copolymers (diBCPs) influence PbBr_2_ dissociation and complexation through surface adsorption in *o*-xylene under stirring. *o*-Xylene is a selective solvent, good for PS but poor for both PEO and PbBr_2_. Five PS_mk_-*b*-PEO_nk_ diBCPs (PS_10k_-*b*-PEO_11.5k_, PS_33k_-*b*-PEO_13.5k,_ PS_33k_-*b*-PEO_35k_, PS_38k_-*b*-PEO_102k_, and PS_140k_-*b*-PEO_141k_) with different block ratios and molecular weights were examined, where the subscripts denote the molecular weights of the constituent blocks in kg mol^–1^. The polymer concentration was fixed at 1 mg/mL. Under this condition, PS-*b*-PEO is interpreted to exist predominantly as dispersed chains with collapsed PEO segments and swollen PS segments, as well as chain-associated clusters, rather than forming well-defined micelles. As a result, surface adsorption is dominated by dispersed chains: PEO segments act as adsorbing blocks through coordination between ether groups and Pb^2+^ cations, while PS segments behave as nonadsorbing blocks swollen by *o*-xylene. Upon addition of PbBr_2_, PS_10k_-*b*-PEO_11.5k_ and PS_33k_-*b*-PEO_13.5k_ effectively induce PbBr_2_ dissociation and complexation to form [Pb_*x*_Br_*y*_]^2*x*−*y*^ complexes, whereas PS_33k_-*b*-PEO_35k_, PS_38k_-*b*-PEO_102k_, and PS_140k_-*b*-PEO_141k_ exhibit a much lower extent of these processes, resulting in negligible complex formation. Two key factors are proposed to account for this behavior. The first is the chain conformation of the adsorbed copolymers. In the proposed adsorption model, PEO blocks adopt train- and loop-like conformations, while PS blocks form tail-like conformations. Short PEO chains favor conformations with a high density of trains and a low density of loops, whereas long PEO chains exhibit the opposite trend. Because PbBr_2_ dissociation and complexation occur at the interface between adsorbed PEO segments and PbBr_2_ surfaces, a higher train density promotes the formation of [Pb_*x*_Br_*y*_]^2*x*−*y*^ complexes. In contrast, long PEO chains favor adsorption conformations dominated by loops with fewer trains, further reducing effective interfacial contact and rendering PbBr_2_ dissociation and complexation sluggish. The second proposed factor is the ability of PS blocks to screen unfavorable PEO–solvent interactions. When PS chains are sufficiently longer than PEO chains, they effectively shield the adsorbed PEO segments from *o*-xylene, thereby stabilizing adsorption and promoting dispersion. In contrast, when PS chains are much shorter than PEO chains, they fail to provide adequate screening, leading to a high interfacial energy penalty that suppresses dispersion and hinders surface adsorption.

## Graphical abstract

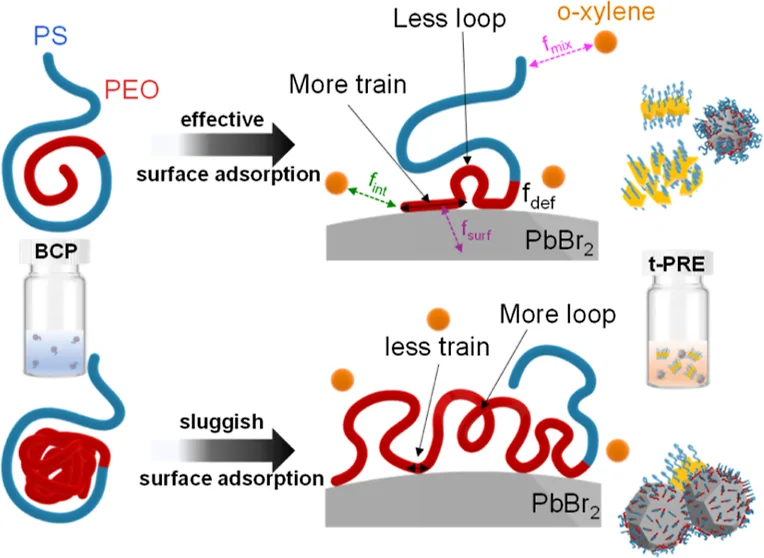

## Introduction

Solution self-assembly of amphiphilic diblock copolymers (diBCPs) has attracted considerable interest because it produces core–shell micelles with diverse morphologies, such as spherical, cylindrical, lamellar micelles, as well as vesicles.^1–8^ These structures arise from the interplay among the stretching of the core-forming block, interactions between the core-forming block and the solvent, and steric repulsion of the shell-forming block.^1^ In contrast to bulk self-assembly,^9^ which is primarily governed by segregation strength in terms of the Flory–Huggins interaction parameter (χ), degree of polymerization, and block volume fractions, solution self-assembly is additionally influenced by solvent quality, which plays a critical role in determining micellar morphology and transitions.^1–8^

Because of their morphological diversity and domain tunability, amphiphilic diBCPs have been widely used as soft templates to direct the growth of inorganic nanomaterials, including metals, metal oxides, semiconductors, and perovskite quantum dots.^10–12^ It is well established that soft-colloidal templating typically involves the addition of precursor salts (e.g., ions or complexes) into solutions containing diBCPs.^10–12^ However, such additives inevitably alter self-assembly behavior. In particular, the presence of ions modifies interchain interactions within the corona, thereby influencing micellar morphology and stability.^1,13,14^ In addition, previous studies have demonstrated that amphiphilic diBCPs containing one crystalline block and one amorphous block can form complex hierarchical hybrid mesostructures when blended with crystallizable additives.^15–36^ These structures arise from the coupling of crystallization and self-assembly (or aggregation), which cannot be achieved by self-assembly alone. These studies demonstrate that additives substantially enrich the morphological diversity and structural complexity of diBCP-additive hybrids. However, the interfacial processes occurring before crystallization and self-assembly remain much less understood, particularly the surface adsorption of diblock copolymers onto inorganic precursor particles. Because surface adsorption governs precursor dissociation, complexation, and subsequent crystallization, understanding how it is affected by the molecular architecture of diblock copolymers remains an important but largely unexplored issue.

For example, our previous studies demonstrated that hybrids of polystyrene-*block*-poly­(ethylene oxide) (PS-*b*-PEO) with PbBr_2_ in selective solvents such as 1,3,5-trimethylbenzene (TMB, good for PS but poor for PEO) or in a neutral solvent such as tetrahydrofuran (THF, good for both PS and PEO) inevitably produce a variety of anisotropic mesostructures, including irregular nanosheets, polygonal nanoplates, compact microplates, seaweed-like mesostructures, and fibers.^37–39^ In particular, irregular nanosheets and polygonal nanoplates serve as intermediate structures during the synthesis of perovskite quantum dots.^40,41^ Notably, these anisotropic one- and two-dimensional mesostructures are not observed in polystyrene-*block*-poly­(2-vinylpyridine) (PS-*b*-P2VP) systems, where the corresponding [Pb_*x*_Br_*y*_]^2*x*−*y*^ complexes are amorphous rather than crystalline and remain confined within the P2VP cores.^42–45^

Our previous studies further demonstrated that the formation of these mesostructures involves multiple processes, including surface adsorption, hierarchical emulsification, PbBr_2_ dissociation, and complexation, interactions between PEO segments and [Pb_*x*_Br_*y*_]^2*x*−*y*^ complexes and finally crystallization-driven self-assembly (CDSA) or inclusion crystallization self-assembly (ICSA) in sequence.^37–39^ Despite these advances, a critical question remains unresolved: how the chemical architecture of block copolymers, particularly molecular weight and block volume fraction, controls surface adsorption and subsequently influences PbBr_2_ dissociation and complexation during multiple emulsification in a selective solvent under stirring. As these processes serve as the initial steps that dictate complex formation, polymorphism, and hierarchical assembly, understanding their dependence on the polymer chain architecture is essential for controlling growth pathways of mesostructures. In this study, we address this question by systematically investigating a series of five PS-*b*-PEO diBCPs with varying molecular weights and block volume fractions in *o*-xylene. By focusing on the dilute polymer concentration employed in this study (1 mg/mL), where surface adsorption is dominated by dispersed chains with collapsed PEO segments but with swollen PS segments, not by molecular micelles, we isolate the role of polymer architecture in controlling interfacial interactions. We propose that PbBr_2_ dissociation and complexation are adsorption-controlled processes that are likely governed by chain conformation and interfacial energetics. In particular, the density of train segments and the ability of PS blocks to screen unfavorable solvent interactions are proposed to regulate the extent of PbBr_2_ dissociation and complexation. These proposed mechanisms provide new insights into the coupling between polymer adsorption and PbBr_2_ dissociation as well as complexation and offer a mechanistic framework for understanding polymorphism and mesoscale assembly in diBCP–additive hybrid systems.

## Experimental Section

Five polystyrene-*block*-poly­(ethylene oxide) diblock copolymers (diBCPs) were purchased from Polymer Source, Inc., and their chemical architectures are summarized in Table 1. For brevity, the notation PS_mk_-*b*-PEO_nk_ is used to denote the diBCPs when their architectures are specified, where mk and nk represent the molecular weights in kg mol^–1^ of the PS and PEO blocks, respectively. The five PS_mk_-*b*-PEO_nk_ diblock copolymers were selected to provide two complementary comparison series. The first series, consisting of PS_10k_-*b*-PEO_11.5k_, PS_33k_-*b*-PEO_35k_, and PS_140k_-*b*-PEO_141k_, was designed to investigate the effect of the overall molecular weight on PbBr_2_ dissociation and complexation through surface adsorption while maintaining approximately symmetric block compositions. The second series, consisting of PS_33k_-*b*-PEO_13.5k_, PS_33k_-*b*-PEO_35k_, and PS_38k_-*b*-PEO_102k_, was designed to investigate the effect of increasing PEO block length (and consequently PEO volume fraction) on PbBr_2_ dissociation and complexation through surface adsorption while maintaining identical or similar PS block molecular weights. PS_33k_-*b*-PEO_35k_ serves as the common reference connecting the two comparison series. Two polystyrene homopolymers (PS_15k_: *M*_n_ = 15 kg mol^–1^, D̵= 1.09; PS_35k_: *M*_n_ = 35 kg mol^–1^, D̵= 1.18; Polymer Source, Inc.) and two poly­(ethylene oxide) homopolymers (PEO_6k_: *M*_n_ = 6 kg mol^–1^; Clariant; and PEO_35k_: *M*_n_ = 35 kg mol^–1^, D̵= 1.08; Polymer Source, Inc.) were used to prepare the control solutions. *o*-Xylene was purchased from Thermo Scientific, and lead­(II) bromide (PbBr_2_, 99.999% trace metals basis, Cat No. 398853) was purchased from Sigma-Aldrich. The PbBr_2_ powder consisted of irregular microparticles with sizes ranging from several to several tens of micrometers. Because of irregular shapes and polydisperse sizes, we did not measure the specific surface area of PbBr_2_. All chemicals were used as received without further purification.

**Table 1:** Molecular Information for the PS-*b*-PEO diBCPs

PS_mk_-*b*-PEO_nk_ with m/n =	*M*_n_^PS^ (kg mol^–1^)	*M*_n_^PEO^ (kg mol^–1^)	*f*_PS_	*f*_PEO_	PDI (D̵)
**10/11.5**	10	11.5	0.48	0.52	1.09
**33/13.5**	33	13.5	0.72	0.28	1.12
**33/35**	33	35	0.50	0.50	1.09
**38/102**	38	102	0.28	0.72	1.10
**140/141**	140	141	0.51	0.49	1.05

In our previous studies,^37,38^ 1,3,5-trimethylbenzene (TMB) was employed as the solvent because its high selectivity toward the PS block facilitates the formation of well-defined complex crystal morphologies, particularly for PS-rich PS-*b*-PEO diblock copolymers. However, the high PS selectivity of TMB also enhances the segregation of the PEO block, thereby facilitating PEO crystallization during prolonged stirring. In addition, TMB provides poor solubility for PEO-rich diblock copolymers (e.g., PS_38k_-*b*-PEO_102k_), making complete dissolution difficult. These two factors may interfere with the adsorption of PS-*b*-PEO chains onto the surfaces of PbBr_2_ microparticles. To minimize these effects and better investigate the adsorption behavior, *o*-xylene, which exhibits a lower solvent selectivity toward the PS block than TMB, was selected in the present study. Although similar complex crystal morphologies are obtained in both solvents, the use of *o*-xylene enables the adsorption process to be examined under conditions where PEO crystallization is less pronounced and both PS-rich and PEO-rich diblock copolymers can be readily dissolved. Furthermore, PS-*b*-PEO diblock copolymers with different molecular weights and block volume fractions possess different critical micelle concentrations (CMCs). At elevated polymer concentrations, they exhibit different degrees of micellization and form distinct micellar morphologies. In addition, higher polymer concentrations promote PEO crystallization in *o*-xylene, particularly for PEO-rich diblock copolymers. Because these factors introduce multiple coupled variables, the present study was intentionally limited to dilute solutions, where well-defined core–shell micelles do not form and the adsorption behavior of PS-*b*-PEO chains can be investigated with fewer interfering effects. To this end, small amounts (1 mg) of PS-*b*-PEO powders were dissolved in 1 mL of *o*-xylene to prepare diBCP solutions. The solutions were sonicated at 70 °C for 30 min to ensure complete dissolution. For simplicity, these solutions are denoted diBCP_*m*/*n*_ when the molecular weights of the PS and PEO blocks are specified. After sonication, 10 mg of PbBr_2_ powder was added to each diBCP solution to prepare turbid precursor solutions. These precursor solutions were stirred at 800 rpm at room temperature for 48 h. For brevity, the turbid precursor solutions are denoted t-PRE_*m*/*n*_, where “t” and “PRE” represent “turbid” and “precursor,” respectively.

The diBCP and t-PRE solutions were first characterized by dynamic light scattering (DLS, Malvern/Zetasizer Nano S) to determine their hydrodynamic diameters (*D*_h_) at the original concentrations. Our previous studies have shown that PbBr_2_ can undergo dissociation and complexation to form [Pb_*x*_Br_*y*_]^2*x*−*y*^ complexes in selective solvents containing PS-*b*-PEO.^37–41^ To probe the formation of these complexes, UV–vis absorbance spectroscopy (JASCO V-770) was performed. Due to detector limitations, the spectra were recorded at one-tenth of the original concentration by diluting small aliquots (∼μL) of the t-PRE solutions with a 10-fold excess of *o*-xylene.

Small-angle and wide-angle X-ray scattering (SAXS/WAXD) measurements were performed at a beamline TPS 13A at the National Synchrotron Radiation Research Center (NSRRC) in Hsinchu. SAXS and WAXD patterns were collected using a beam wavelength of λ = 0.826 Å with Eiger X 9 M and Eiger X 1 M detectors under vacuum, respectively. For these measurements, diBCP and t-PRE aliquots were sealed in quartz capillary tubes (diameter = 2 mm). Based on Orion et al.’s study,^46^ background subtraction and data reduction were carried out to obtain one-dimensional SAXS and WAXD profiles with absolute intensity as a function of the scattering vector q, where *q* = (4π/λ) sinθ and θ is the scattering angle. SAXS curves were fitted using the SasView software.

To examine the morphologies, transmission electron microscopy (TEM, Hitachi H-7500) was employed. Dried samples were prepared by drop-casting aliquots of diBCP or t-PRE solutions onto TEM grids, with filter paper placed underneath to facilitate the rapid removal of *o*-xylene, followed by drying in a vacuum oven overnight. TEM images were acquired at an acceleration voltage of 60 kV. The dried samples were also characterized by low-voltage TEM (LV-TEM, JEOL JEM-1400), electron diffraction (ED), and energy-dispersive spectroscopy (EDS) to investigate the structural and chemical characteristics of the products in the t-PRE_*m*/*n*_ solutions. All of these characterizations were performed at an accelerating voltage of 120 kV. Atomic force microscopy (AFM100plus, Hitachi) was performed to characterize the surface morphologies of dried samples prepared from diBCP_*m*/*n*_ solutions.

For comparison, five control samples were prepared by dispersing 10 mg of PbBr_2_ in (i) polymer-free *o*-xylene, (ii), (iii) *o*-xylene containing 1 mg/mL PEO (PEO_6k_ and PEO_35k_), and (iv), (v) *o*-xylene containing 1 mg/mL PS (PS_15k_ and PS_35k_). All control samples were stirred at 800 rpm for 48 h under the same conditions used for the PS-*b*-PEO systems. In these control solutions, PbBr_2_ exhibited poor dispersion and inevitably precipitated during stirring. After stirring, the precipitates were collected for in-house wide-angle X-ray diffraction (WAXD, Rigaku SmartLab) characterization, which was performed at 40 kV and 30 mA. WAXD profiles were collected at a scan rate of 50° min^–1^ using Cu Kα radiation. For comparison, neat PEO_6k_ crystallites were also prepared under the same conditions (800 rpm, 48 h) and characterized by in-house WAXD.

## Results and Discussion

### Self-Assembly Behavior of Neat PS-*b*-PEO diBCPs in *o*-Xylene

The freshly prepared diBCP solutions appeared transparent with no observable Rayleigh scattering or Tyndall effect (Figure 1A). This observation indicates that PS-*b*-PEO chains do not form micellar structures at 1 mg/mL.

**Figure 1:**
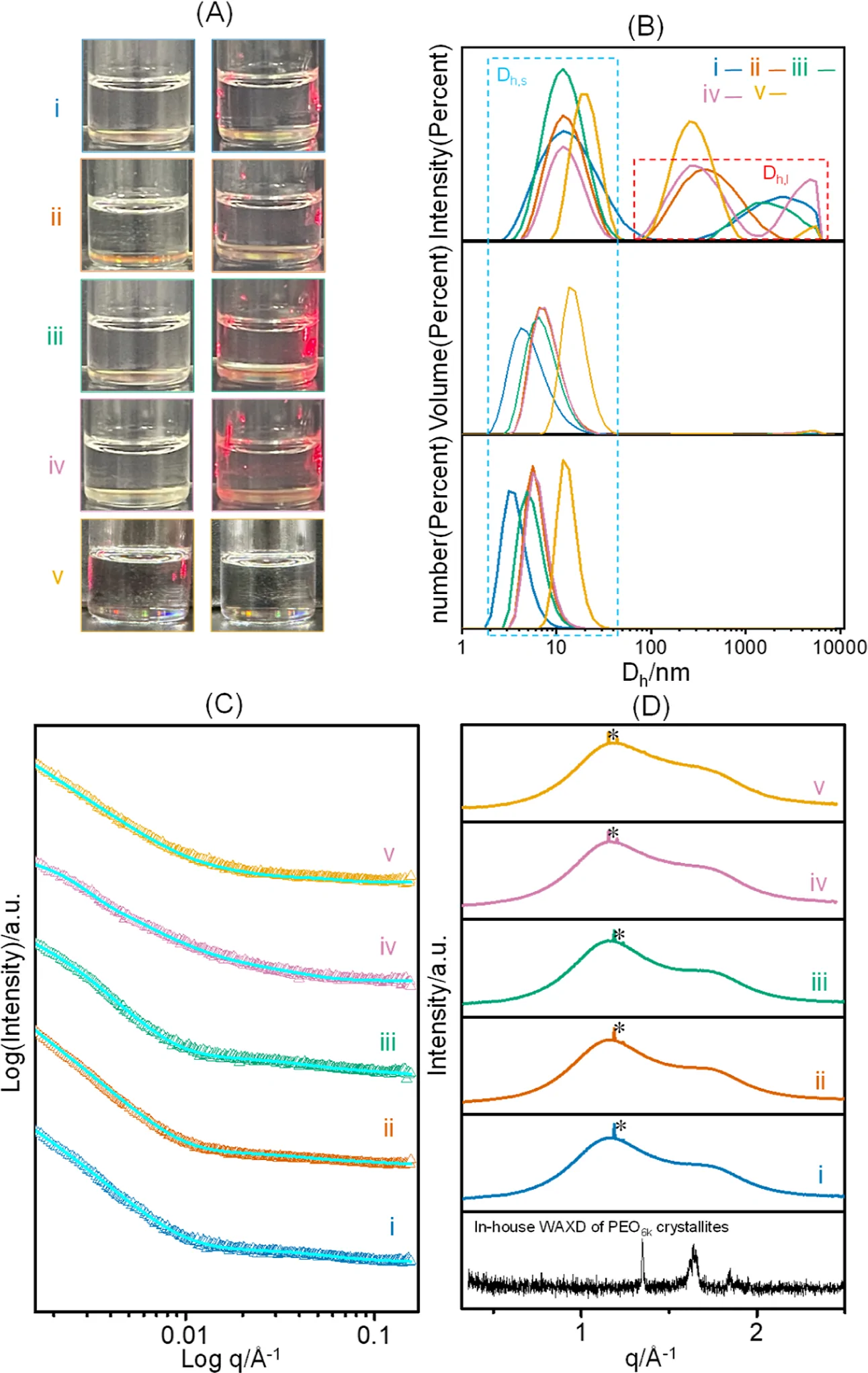
(A) Photos, (B) DLS profiles, (C) SAXS profiles (symbols: experimental data; lines: fitted curves), and (D) WAXD profiles of diBCP_*m*/*n*_ solutions with *m*/*n* = (i) 10/11.5, (ii) 33/13.5, (iii) 33/35, (iv) 38/102, and (v) 140/141. In (A), Tyndall tests were performed by exposing the solutions with a red-laser beam. The bottom inset in (D) shows the in-house WAXD profile of monoclinic PEO_6k_ crystallites. In (D), the asterisks denote unknown diffraction peaks arising from impurities. For visual guidance, all the SAXS and WAXD curves are vertically shifted.

Nevertheless, intensity-weighted dynamic light scattering (DLS) profiles exhibit bimodal or trimodal size distributions (the top panel of Figure 1B). Regardless of chemical architecture, most of the diBCP solutions display a primary size distribution centered at *D*_h,s_ ∼10 nm. In contrast, the peaks at larger hydrodynamic diameters (*D*_h,l_) show no systematic trend and vary irregularly. Moreover, such multimodal distributions are absent in the volume- and number-weighted DLS profiles; instead, only one peak corresponding to *D*_h,s_ is present (the middle and bottom panels of Figure 1B).

It is well established that DLS intensity is strongly dependent on the particle size, with the scattering intensity scaling approximately with the sixth power of particle size, whereas volume-weighted distributions scale with the third power.^47^ Therefore, even a small fraction of large aggregates can produce apparent large-*D*_h_ peaks in intensity-weighted profiles.

These results indicate that larger structures, if present, exist only in minor amounts. In other words, the diBCP solutions are dominated by small species with an average size of *D*_h,s_ ∼10 nm. Two concerns deserve further consideration. The first concern is that the absence of a visible Tyndall effect together with the DLS results is also consistent with the presence of other small association species. In other words, the existence of very small or transient association species cannot be completely excluded based on the DLS measurements alone. Species such as single-chain micelles, flower-like micelles, or other small polymer associations with characteristic dimensions below approximately 10 nm may exhibit similar hydrodynamic sizes. However, according to previous studies, single-chain micelles are predominantly reported for dendrimers, hyperbranched polymers, multiarm star polymers, and cyclic polymers,^48^ whereas flower-like micelles are typically observed in ABA triblock copolymers, telechelic polymers, or polymers bearing bulky end groups.^49–52^ To the best of our knowledge, such structures have not been widely reported for linear diblock copolymers under conditions similar to those employed in the present study. Nevertheless, consistent with our interpretation, Bhargava et al.^6^ observed a small DLS peak at approximately 10 nm for linear PS-*b*-PEO and attributed it to unassociated PS-*b*-PEO single chains rather than well-defined micelles.

The second concern involves the inherent limitations of DLS analysis. Commercial DLS analysis assumes spherical particles, whereas the volume- and number-weighted distributions are mathematically transformed from the intensity-weighted distribution.^47,53^ Consequently, for ill-defined or nonspherical particulates, the converted size distributions are subject to additional uncertainty and should be interpreted qualitatively rather than quantitatively. Moreover, if dilute solutions contain ill-defined or nonspherical particulates, DLS alone is generally insufficient to unambiguously distinguish among different association species without complementary structural characterization, such as SAXS or SANS.^53^

To verify structural details, the diBCP solutions were characterized by SAXS and WAXD. The SAXS profiles, presented in absolute intensity as a function of *q*, exhibit similar scattering features with intensities well below ∼10 cm^–1^ (Figure S1). Because portions of the SAXS curves overlap, vertically shifted profiles are shown in Figure 1C to better visualize subtle differences in the scattering features. As shown in Figure 1C, all SAXS profiles exhibit a low-q upturn at *q* < 1 Å^–1^, followed by a plateau at *q* > 1 Å^–1^. The low-q upturn shows an intensity decay following I ∼ q^–α^ (α ≈ 3.1–3.6), whereas the high-q region displays nearly q-independent intensity. The corresponding WAXD profiles show only broad diffuse halos, which are attributed to inter- and intrachain correlations (Figure 1D). No diffraction peaks corresponding to monoclinic PEO crystals were observed for the diBCP_*m*/*n*_ solutions, whereas such peaks are clearly present for the PEO_6k_ homopolymer (see the bottom inset of Figure 1D). The absence of distinct diffraction peaks corresponding to monoclinic PEO crystals (*a* = 8.05 Å, *b* = 13.04 Å, *c* = 19.48 Å; β = 125.4°; *P*2_1_/*a*)^54^ indicates that no PEO crystallites are formed in the diBCP solutions. Therefore, the low-q upturn is unlikely to originate from crystalline domains and is instead interpreted as being consistent with the presence of chain clusters.

To investigate the structural origin of these small and large species, the SAXS profiles were fitted using a two-level Beaucage model. The two-level Beaucage model was selected because the SAXS profiles (Figure 1C) exhibit hierarchical scattering over two characteristic length scales, making the model suitable for empirically describing the observed structural hierarchy without assuming a specific particle geometry.^55–57^ The two-level model therefore provided satisfactory fits to the experimental SAXS profiles with χ^2^ values of 1 ∼ 2 and small fitting residuals (see Table S1 and Figure S2). The fitting parameters are summarized in Table S1. The radius of gyration (*R*_g,s_) of the small structural level ranges from ∼3.6 to ∼6.3 nm, while that of the larger structural level ranges from ∼116 to ∼240 nm. The large structural level exhibits fractal dimensions of ∼3–4, indicating relatively compact structures. Notably, no precipitation was observed even after prolonged incubation, suggesting that these larger aggregates remain stably dispersed in solution. Although a large structural level (*R*_g,l_ ≈ 116–240 nm) is identified with the Beaucage analysis, the solutions remain optically transparent without an observable Tyndall effect (see the right column of Figure 1A). This is likely due to the extremely low number density of these large aggregates and the small refractive index contrast between PS-*b*-PEO and *o*-xylene, resulting in negligible light scattering at the macroscopic scale.

To further examine the dried-state morphology, TEM and AFM characterizations were performed on samples prepared by drop-casting the diBCP solutions. As shown in Figures 2 and S3, the dried samples predominantly exhibit continuous low-contrast polymer features together with a small number of locally thickened regions. No well-defined spherical nanodomains or core–shell micellar structures are observed. Because the drying process may alter the original solution morphology, these TEM and AFM observations are interpreted as only supporting evidence. Nevertheless, the absence of well-defined micellar particles is generally consistent with the DLS and SAXS results, which indicate that well-defined core–shell micelles were not detected under the present experimental conditions.

**Figure 2:**
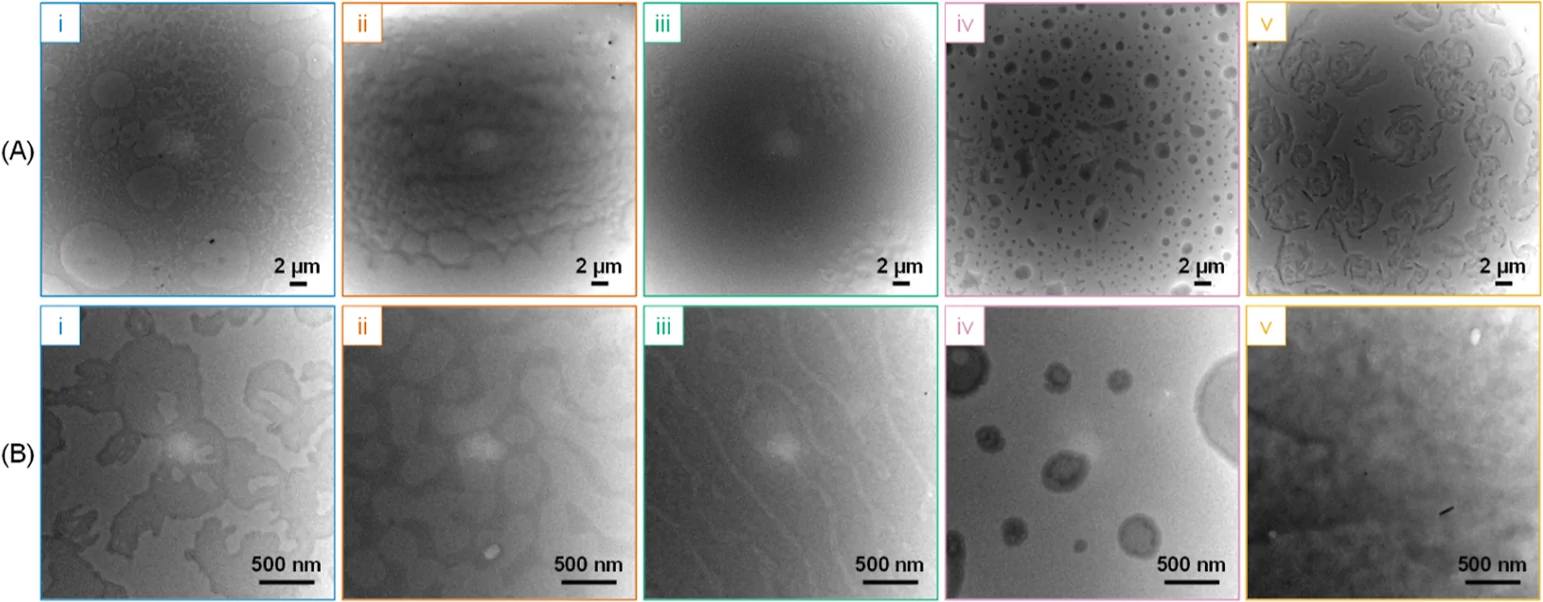
(A) Low- and (B) high-magnification TEM images of diBCP_*m*/*n*_ dried samples with *m*/*n* = (i) 10/11.5, (ii) 33/13.5, (iii) 33/35, (iv) 38/102, and (v) 140/141.

On the basis of the combined DLS, SAXS, WAXD, TEM, and AFM results, we propose that neat PS-*b*-PEO block copolymers predominantly exist as dispersed chains, accompanied by a minor fraction of chain-associated clusters in *o*-xylene (Figure 3). The proposed model is based on previous studies.^7,8^ Within this proposed structural model, dispersed polymer chains constitute the predominant species, whereas chain-associated clusters are interpreted to represent only a minor population. No core–shell micelles were detected under these conditions. If abundant core–shell micelles with characteristic dimensions of several tens of nanometers were present, then more pronounced Rayleigh scattering and Tyndall effects would generally be expected. Therefore, under the experimental conditions employed, the polymer concentration of 1 mg/mL did not produce detectable well-defined core–shell micelles.

**Figure 3:**
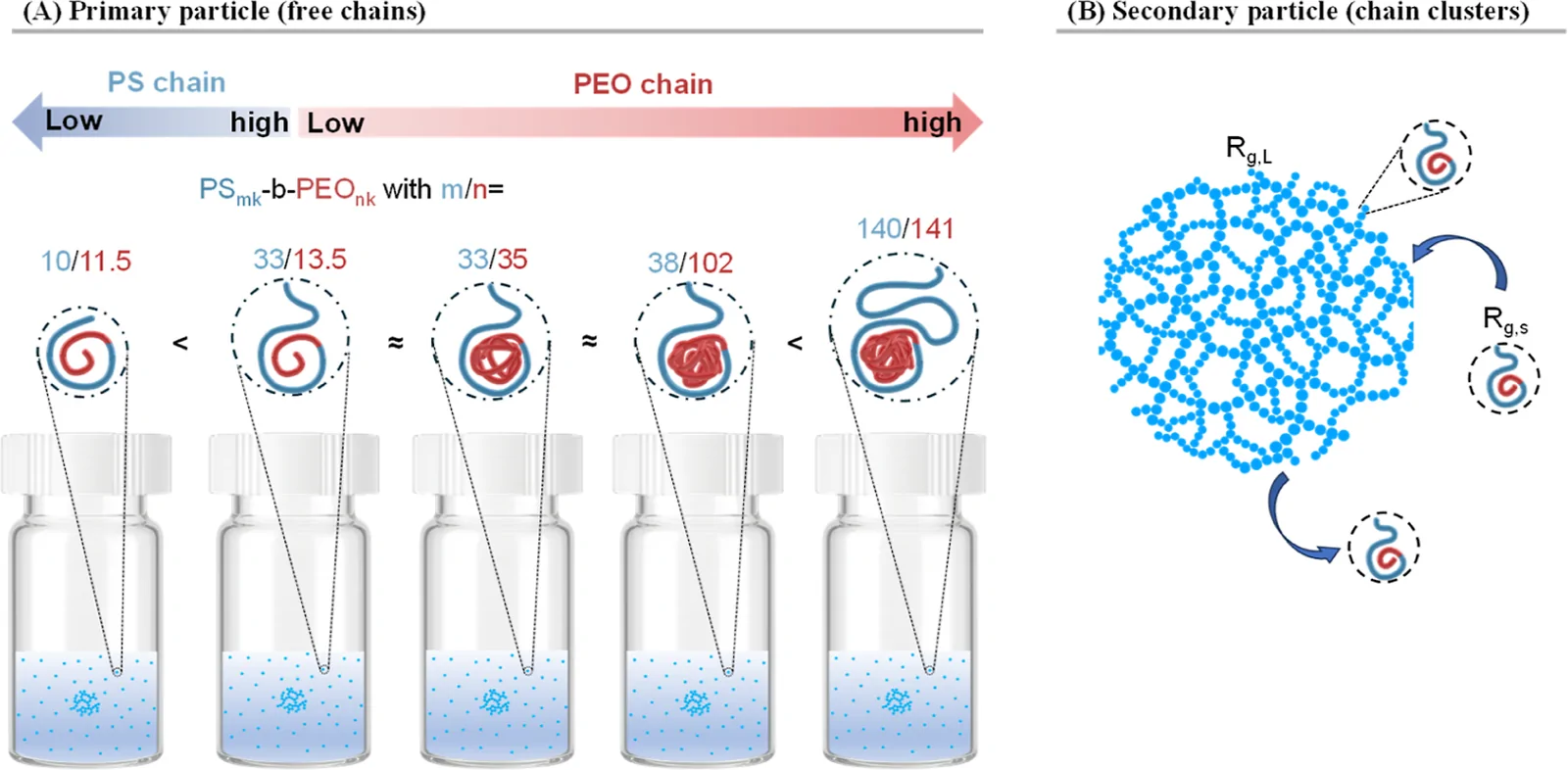
Schematic illustration of free chains and chain clusters in diBCP solutions prepared by dissolving PS-*b*-PEO of various chemical architectures in *o*-xylene.

*o*-Xylene is a selective solvent (solubility parameter: δ = 18 MPa^1/2^),^58^ being good for PS (δ = 19.2 MPa^1/2^)^7^ but poor for PEO (δ = 20.6 MPa^1/2^). Thus, as shown in Figure 3, when PS-*b*-PEO exists as dispersed chains, the two blocks adopt distinct conformations: PEO segments collapse into compact cores, while PS segments extend into the solvent (Figure 3). The collapse of PEO segments minimizes unfavorable interactions with *o*-xylene, whereas the surrounding PS chains partially shield the PEO cores from the solvent.

DLS measurements show that the small species exhibit similar *D*_h,s_ values, independent of the molecular weight and block volume fraction (Figure 1B). This is consistent with the possibility that even in copolymers with a high PEO content, such as PS_38k_-*b*-PEO_102k_, the PEO blocks adopt highly compact conformations. Otherwise, the relatively short PS blocks would not be able to effectively screen the PEO domains. On the basis of this consideration, we propose that the PEO cores in PS_38k_-*b*-PEO_102k_ are more compact than those in the other copolymers.

To support this interpretation, we theoretically estimate the radius of gyration (*R*_g_^0^) of unperturbed PS-*b*-PEO chains following the procedure described in the Supporting Information. The calculated (*R*_g_^0^) values are summarized in Table 2, together with the *D*_h,s_ and *R*_g,s_ values obtained experimentally from DLS and SAXS, respectively. As shown in Table 2, the theoretical (*R*_g_^0^) increases with increasing molecular weights of the PS and PEO blocks. However, this trend is not observed for the experimentally measured *D*_h,s_ and *R*_g,s_. In particular, significant deviations between experimental and theoretical *R*_g_ values are found for copolymers with longer PEO blocks. This discrepancy suggests that PEO chains undergo pronounced contraction in *o*-xylene (Figure 3A). Although such chain shrinkage incurs an entropic penalty, it is compensated by a reduction in enthalpic penalty arising from the avoidance of unfavorable PEO–solvent interactions.

**Table 2:** A Summary of *D*_h,s_, *R*_g,s_, and *R*_g_^0^ Obtained for the Used PS_mk_-B-PEO_nk_ diBCPs

PS_mk_-*b*-PEO_nk_ with *m*/*n* =	*N*_PS_	*N*_PEO_	*D*_h,s_ (nm)	*R*_g,s_ (nm)	*R*_g_^o^ (nm)
**10/11.5**	96	261	10	3.60	4.87
**33/13.5**	316	306	10	4.67	6.61
**33/35**	316	794	10	4.43	8.61
**38/102**	364	2315	10	5.13	13.06
**140/141**	1344	3200	17	6.30	17.40

In addition, when the PS blocks are too short to effectively shield the collapsed PEO domains, interchain association is expected to become more favorable, leading to interchain clustering (Figure 3B). However, clustering is not governed solely by block length. Owing to strong Brownian motion at this length scale, PS-*b*-PEO chains continuously fluctuate and PS segments cannot maintain complete coverage of the PEO domains. This may result in transient exposure of PEO segments to the solvent, allowing unfavorable PEO–solvent interactions to persist and thereby facilitating interchain association.

Consistent with the proposed model, these chain-associated clusters exhibit broad size distributions. In particular, the high-*D*_h_ peaks in the intensity-weighted DLS profiles vary with the measurement time, whereas the low-*D*_h_ peaks remain nearly constant. This behavior suggests that the larger aggregates are transient in nature and present only in minor amounts.

### Hybrids of PS-*b*-PEO diBCPs and PbBr_2_ in *o*-Xylene

In the following, we demonstrate that the extent of PEO shrinkage, governed by the relative lengths of the PEO and PS blocks, influences the degree of PbBr_2_ dissociation and complexation, multiple emulsification, particle dispersion, and ultimately mesocrystal formation in *o*-xylene-containing PS-*b*-PEO with distinct chemical architectures.

After 10 mg of PbBr_2_ powder was added to the diBCP solutions, the mixtures were stirred at 800 rpm at room temperature for 48 h to prepare fresh t-PRE solutions. The t-PRE solutions were characterized by visual observations and the Tyndall effect to evaluate particle dispersion and precipitation; by UV–vis absorbance spectroscopy to probe PbBr_2_ dissociation and complex formation; by DLS and SAXS to characterize hierarchical particle structures in solution; and by WAXD, TEM, and ED to identify crystalline phases and examine the morphology of the resulting hybrid particles.

The freshly prepared t-PRE solutions appeared to be turbid and exhibited strong diffuse scattering, rendering the Tyndall effect difficult to observe (Figure 4A). Notably, the turbidity is transient for PS_33k_-*b*-PEO_35k_, PS_38k_-*b*-PEO_102k_, and PS_140k_-*b*-PEO_141k_. After 24 h of static incubation following the cessation of stirring, the corresponding t-PRE_33/35_, t-PRE_38/102_, and t-PRE_140/141_ solutions became transparent due to precipitation (Figure S4). In contrast, the turbidity remained relatively stable for PS_10k_-*b*-PEO_11.5k_ and PS_33k_-*b*-PEO_13.5k_, and little precipitation was observed after 24 h of static incubation for t-PRE_10/11.5_ and t-PRE_33/13.5_, rendering the Tyndall effect easy to observe.

**Figure 4:**
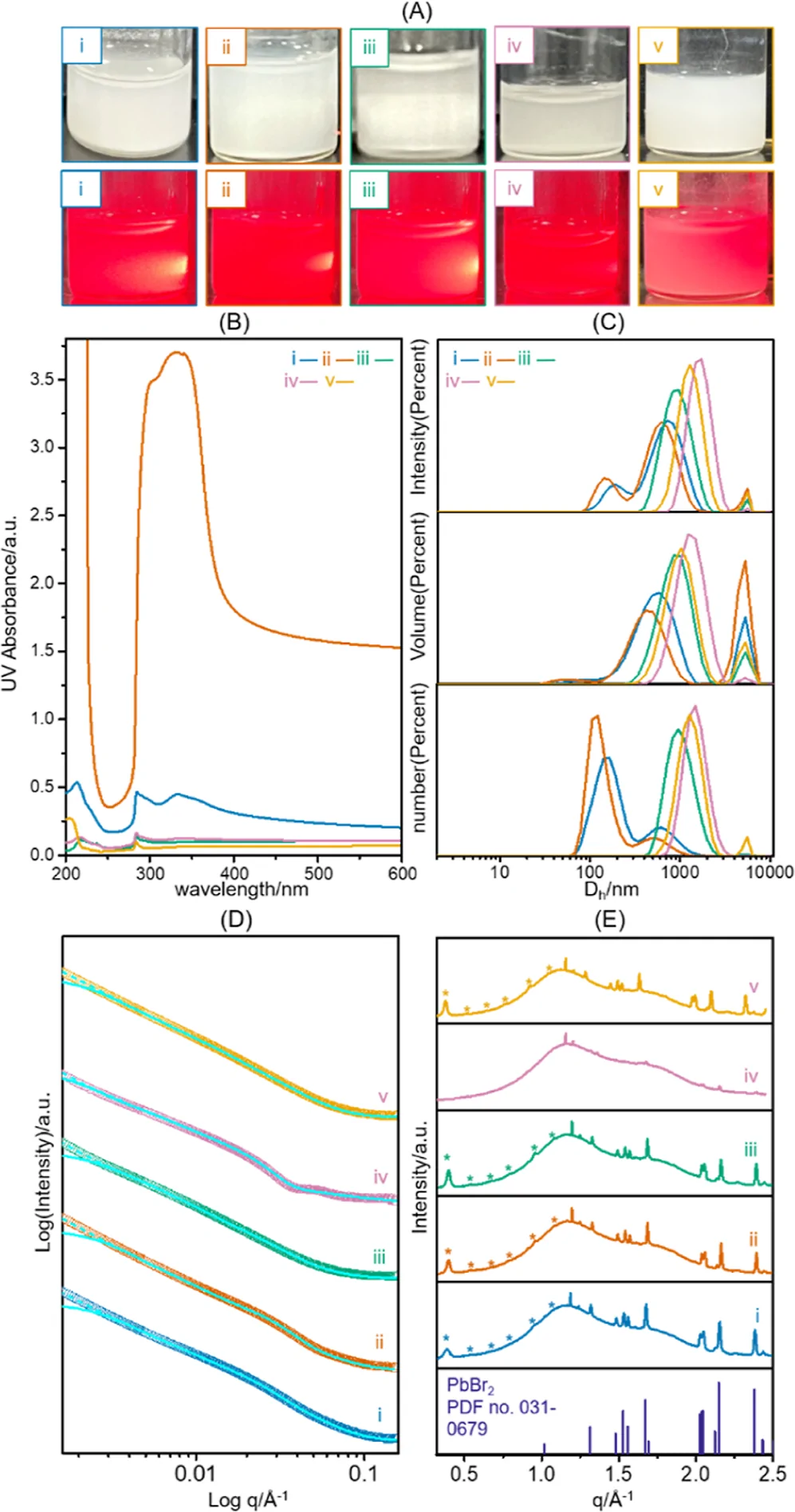
(A) Photos, (B) UV–vis absorbance spectra, (C) DLS profiles, (D) SAXS profiles (symbols: experimental data; solid lines: fitted curves), and (E) WAXD profiles of freshly prepared t-PRE_*m*/*n*_ solutions with *m*/*n* = (i) 10/11.5, (ii) 33/13.5, (iii) 33/35, (iv) 38/102, and (v) 140/141. In (A), Tyndall tests were performed by exposing the solutions with a red-laser beam. The stick pattern in (E) represents the standard WAXD profile of orthorhombic PbBr_2_ crystals with a *Pnma* symmetry (PDF #031–0679). In (E), the reflections of the orthorhombic complex crystal are highlighted by asterisks.

Furthermore, distinct chemical architectures of PS-*b*-PEO lead to markedly different extents of [Pb_*x*_Br_*y*_]^2*x*−*y*^ complex formation via diBCP-assisted PbBr_2_ dissociation and complexation. UV–vis absorbance spectra show that copolymers with shorter PEO blocks and longer PS blocks promote a higher concentration of [Pb_*x*_Br_*y*_]^2*x*−*y*^ complexes, whereas those with longer PEO blocks and shorter PS blocks exhibit minimal or negligible complex formation (Figure 4B). These [Pb_*x*_Br_*y*_]^2*x*−*y*^ complexes give rise to broad absorption over the 300–400 nm region, consistent with previous studies.^37–41^

Figure 4C shows the corresponding DLS profiles. As shown, all t-PRE solutions exhibit multiple broad peaks with hydrodynamic diameters (*D*_h_) ranging from ∼100 nm to several micrometers in the intensity-, volume-, and number-weighted distributions. This result indicates that the t-PRE solutions contain dispersed particles with a broad size distribution. Notably, the sharp peak at *D*_h,s_ ≈ 10 or 17 nm observed in the diBCP solutions disappears upon addition of PbBr_2_, suggesting that the original dispersed chains or small clusters are incorporated into dispersed particles during stirring.

The number-weighted DLS profiles further reveal that PS_10k_-*b*-PEO_11.5k_ and PS_33k_-*b*-PEO_13.5k_ promote fragmentation of PbBr_2_ microparticles, resulting in a higher population of smaller dispersed particles in the t-PRE_10/11.5_ and t-PRE_33/13.5_ solutions. In contrast, PS_33k_-*b*-PEO_35k_, PS_38k_-*b*-PEO_102k_, and PS_140k_-*b*-PEO_141k_ are less effective in fragmenting PbBr_2_ microparticles, leading to the persistence of larger particles.

Figure 4D shows the SAXS profiles of the fresh t-PRE solutions, which exhibit multiple power-law scattering regimes across different *q* regions. No diffraction peaks are observed, indicating the absence of long-range spatial ordering among the dispersed particles. All samples display a pronounced low-*q* upturn at *q* < 0.004 Å^–1^, suggesting the presence of large-scale structures or aggregates. The absence of well-defined form-factor oscillations further indicates that these structures are highly polydisperse and lack uniform shapes.

The absolute-intensity SAXS profiles (Figure S5) reveal that the t-PRE_*m*/*n*_ solutions exhibit scattering intensities several orders of magnitude higher than those of the corresponding diBCP_*m*/*n*_ solutions (Figure S1). This enhanced scattering originates primarily from the addition of PbBr_2_, owing to the large electron density contrast associated with the Pb and Br elements. Furthermore, the t-PRE_10/11.5_ and t-PRE_33/13.5_ solutions display the highest absolute intensities, followed by t-PRE_33/35_ and t-PRE_140/141_, whereas t-PRE_38/102_ exhibits the lowest intensity.

We further fitted the SAXS curves using the two-level Beaucage model. Using this model, the dimensionality exponent (α) and radii of gyration (*R*_g_) corresponding to particulate structures at different length scales were extracted. The fitted SAXS curves were generated using the structural parameters summarized in Table S2. No clear overall trend in the structural size was identified, likely due to pronounced size polydispersity, structural heterogeneity, and differences in dispersion stability among the t-PRE solutions. Nevertheless, Table S2 suggests that PS_10k_-*b*-PEO_11.5k_ and PS_33k_-*b*-PEO_13.5k_ favor the formation of relatively small secondary particles (*R*_g,l_ ≈ 70.3 and 66.8 nm), whereas PS_33k_-*b*-PEO_35k_, PS_38k_-*b*-PEO_102k_, and PS_140k_-*b*-PEO_141k_ tend to form larger secondary particles (*R*_g,l_ ≈ 141.2, 156.2, and 89.2 nm). It should be noted that the two-level Beaucage model cannot adequately fit the low-*q* region (*q* < 0.003 Å^–1^) of the SAXS curves, where pronounced low-*q* intensity upturns dominate. This discrepancy results in high χ^2^ values and large residuals (Table S2 and Figure S6). Closer examination of the low-*q* upturns highlighted by dotted lines in Figure 4D indicates that the scattering intensity follows an *I* (*q*) ∝ *q* ^–α^ dependence (α > 3.5). Chung et al. have demonstrated that these pronounced low-*q* upturns originate from PbBr_2_ microparticles.^40^ As the PbBr_2_ microparticles are removed by centrifugation, the pronounced low-*q* upturn is suppressed.

Figure 4E shows the WAXD profiles. Two prominent features are observed. First, all t-PRE solutions display a series of diffraction peaks (*q* = 1.080, 1.317, 1.485, 1.533, 1.565, 1.676, 2.035, 2.049, 2.131, 2.158, 2.385, and 2.437 Å^–1^) at *q* > 1 Å^–1^, which can be indexed to (110), (020), (011), (120), (200), (111)/(210), (121)/(220), (201), (130), (211), (031), and (310)/(221) reflections of orthorhombic PbBr_2_ crystals with a *Pnma* symmetry, respectively. These diffraction peaks indicate the presence of crystalline PbBr_2_ in all t-PRE solutions. The diffraction peaks are noticeably weaker for the t-PRE_38/102_ sample than for the other four samples.

Second, except for the t-PRE_38/102_ solution, the other four samples exhibit additional weak diffraction peaks (indicated by asterisks) at *q* < 1 Å^–1^. These reflections can be assigned to orthorhombic complex crystals, with the most intense peak at *q* ≈ 0.39 Å^–1^ corresponding to the (002) reflection. The remaining peaks at *q* ≈ 0.537, 0.674, 0.793, 0.954, and 1.080 Å^–1^ are assigned to (200), (202), (004), (204), and (400) reflections of the orthorhombic complex crystals, respectively, based on previous studies.^37^ These additional low-*q* reflections are observed for the t-PRE_10/11.5_, t-PRE_33/13.5_, t-PRE_33/35_, and t-PRE_140/141_ samples but are absent in the t-PRE_38/102_ sample. Notably, the characteristic low-q diffraction peaks assigned to the orthorhombic complex crystals are absent in the control samples containing only PbBr_2_, PbBr_2_/PEO, or PbBr_2_/PS in *o*-xylene (Figure S7).

Figure 5 shows TEM images of dried samples prepared from freshly prepared t-PRE_*m*/*n*_ solutions. Three representative morphologies were observed. First, irregular nanosheets are the dominant morphology in the t-PRE_33/13.5_ sample, although they coexist with PbBr_2_ microparticles and polygonal nanoplates as minor components (Figure 5A (ii),B (ii)). Second, the t-PRE_33/35_, t-PRE_38/102_, and t-PRE_140/141_ samples are dominated by PbBr_2_ microparticles, with irregular nanosheets being scarcely observed (Figure 5A (iii–v),B (iii–v)). Third, the t-PRE_10/11.5_ sample exhibits an intermediate morphology between these two extremes (Figure 5A (i),B (i)). Although both t-PRE_33/13.5_ and t-PRE_10/11.5_ samples contain numerous irregular nanosheets, a subtle difference is noted: in t-PRE_33/13.5_, the nanosheets are well dispersed and uniformly distributed (Figure 5A (ii),B (ii)), whereas in t-PRE_10/11.5_ they tend to cluster around PbBr_2_ microparticles (Figure 5A (i),B (i)).

**Figure 5:**
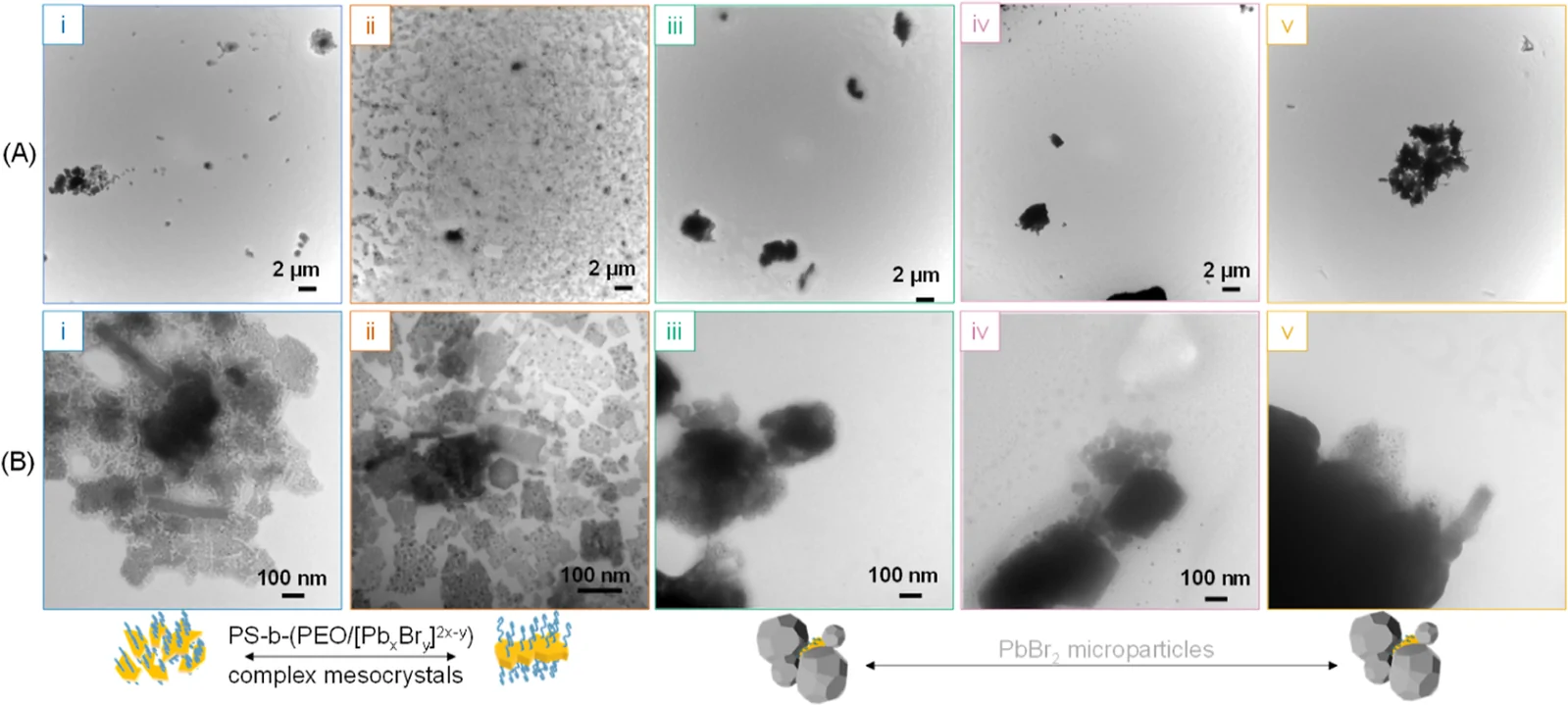
(A) Low- and (B) high-magnification TEM images of dried samples prepared by drop-casting aliquots of freshly prepared t-PRE_*m*/*n*_ solutions with *m*/*n* = (i) 10/11.5, (ii) 33/13.5, (iii) 33/35, (iv) 38/102, and (v) 140/141.

Our previous studies have demonstrated that these irregular nanosheets consist of orthorhombic complex crystals formed via diBCP-assisted PbBr_2_ dissociation and complexation.^37–44^ To identify the structural and chemical characteristics of the products, irregular nanosheets and PbBr_2_ microparticles were further characterized by LV-TEM, ED, and EDS. As shown in Figure S8A,B, the irregular nanosheets formed in the t-PRE_10/11.5_ and t-PRE_33/13.5_ solutions exhibit single-crystal electron diffraction (ED) patterns. The diffraction spots can be indexed to the (*hk*0) reflections of the orthorhombic complex crystal (*a* = 23.53 Å, *b* = 4.20 Å, *c* = 33.22 Å; α = β = γ = 90°) with the electron beam oriented along the [001] zone axis. In contrast, the PbBr_2_ microparticles formed in the t-PRE_33/35_, t-PRE_38/102_, and t-PRE_140/141_ solutions do not exhibit well-defined single-crystal ED patterns. Only very weak diffraction spots are occasionally observed, making reliable indexing impossible. The weak diffraction intensity is likely attributable to the relatively large thickness of the PbBr_2_ microparticles, which strongly attenuates the electron beam.

Furthermore, the EDS spectra exhibit characteristic peaks corresponding to C, O, Pb, and Br (Figure S8C). In the irregular nanosheets, the coexistence of these elements is consistent with the incorporation of PS-*b*-PEO chains into the hybrid complex crystals. However, the uncomplexed PbBr_2_ microparticles also exhibit C, O, Pb, and Br signals despite the absence of hybrid complex crystals. A representative PbBr_2_ microparticle was further analyzed by comparing EDS spectra acquired from the particle edge and interior (Figure S9). The edge region exhibits markedly stronger carbon and oxygen signals, whereas the particle interior is dominated by Pb and Br signals, with only weak carbon and oxygen signals. Together with the observed thin, low-contrast surface layer, these observations are consistent with the presence of a PS-*b*-PEO-rich surface layer and provide supporting evidence for the surface adsorption of PS-*b*-PEO chains onto PbBr_2_ microparticles.

On the basis of the relative populations of irregular nanosheets and PbBr_2_ microparticles observed in the TEM images (Figure 5), we propose that the degree of PbBr_2_ dissociation and complexation follows the order: t-PRE_33/13.5_ and t-PRE_10/11.5_ ≫ t-PRE_33/35_, t-PRE_38/102_, and t-PRE_140/141_ (Figure 5). This trend is consistent with that inferred from the UV–vis absorbance spectra (Figure 4B).

Furthermore, PbBr_2_ microparticles exhibit a poor dispersion in certain systems. After 24 h of static aging, most PbBr_2_ microparticles precipitate in the t-PRE_33/35_, t-PRE_38/102_, and t-PRE_140/141_ solutions, leaving primarily PS-*b*-PEO chains in the supernatants. The primarily PS-*b*-PEO chains further crystallized as PS-*b*-PEO mesocrystals (Figure S10A (iii–v),B (iii–v)). In contrast, the t-PRE_10/11.5_ and t-PRE_33/13.5_ solutions behave differently. Because of the higher extent of PbBr_2_ dissociation and complexation, some PbBr_2_ microparticles fragment into irregular nanosheets (major) and polygonal nanoplates (minor). These mesostructures exhibit relatively good dispersion and remain in the supernatants even after 24 h of static aging (Figure S10A (i,ii),B (i,ii)).

It should be noted that all t-PRE_*m*/*n*_ solutions were prepared under identical experimental conditions so that the effects of the PS-*b*-PEO molecular weight and block volume fraction on PbBr_2_ dissociation and complexation through surface adsorption could be isolated. Therefore, the observed mesoscale morphologies represent the intrinsic products formed under identical preparation conditions rather than structures obtained through a morphology-controlled synthesis.

### Proposed Mechanism for PbBr_2_ Dissociation and Complexation

In our system, at a low concentration of 1 mg/mL PS-*b*-PEO in *o*-xylene, DLS and SAXS did not detect the formation of well-defined core–shell micelles. Instead, PS-*b*-PEO is interpreted to exist predominantly as dispersed polymer chains together with ill-defined clusters. These clusters are likely transient and form to minimize unfavorable contacts between PEO and *o*-xylene. Because *o*-xylene is a selective solvent (good for PS but poor for PEO), long PEO chains are likely to contract, allowing them to be partially shielded by PS segments. If the PS blocks are not sufficiently long to effectively screen PEO from the solvent, further contraction is expected to be favored. Although this contraction incurs an entropic penalty, it is compensated by a reduction in the enthalpic penalty associated with unfavorable PEO–solvent interactions.

Upon the addition of PbBr_2_ in the presence of PS-*b*-PEO in *o*-xylene, surface adsorption of PS-*b*-PEO chains onto PbBr_2_ microparticles may occur. PbBr_2_ is an orthorhombic crystal whose surfaces are enriched with exposed Pb^2+^ cations and contain fewer Br^–^ anions than the bulk crystal. As a result, the surface Pb^2+^ cations are coordinatively unsaturated and preferentially coordinate with electron-rich molecules, such as the ether oxygen atoms of the PEO blocks, via the Lewis acid–base interaction.^59,60^ However, whether effective or sluggish adsorption takes place likely depends strongly on the relative chain lengths of the PS and PEO blocks. Furthermore, the following discussion focuses on interfacial adsorption because the key processes investigated in the present study, namely, PbBr_2_ dissociation and the subsequent formation of [Pb_*x*_Br_*y*_]^2*x*−*y*^ complexes, are expected to occur primarily at the polymer/PbBr_2_ interface. Therefore, the existence of interfacial adsorption, rather than the exact number of adsorbed polymer layers, is considered to be the key factor governing the proposed mechanism.

Motschmann et al. demonstrated that adsorption of PS-*b*-PEO onto solid surfaces proceeds through a two-stage process, involving an initial diffusion-controlled adsorption stage followed by chain penetration and conformational rearrangement within the adsorbed layer.^61^ Furthermore, Munch et al. demonstrated that surface adsorption from micelles is quicker than surface adsorption from free chains.^62^ Johner et al. demonstrated that surface adsorption from a micelle phase, where BCP concentrations are above the CMC in a selective solvent, involves the release of free chains from a micelle, then surface adsorption of released free chains, and finally adsorption constraints resulting from dense layers of adsorbed chains.^63^ Such multiple processes result in three regimes with different adsorption kinetics.

In the present system, however, stirring induces strong convective transport, which dominates over molecular diffusion, in agreement with our previous study.^64^ Therefore, the molecular weight dependence of diffusion from the liquid medium to the solid surface can be neglected. Here, we focus on the effects of conformations of adsorbed chains on effectiveness of PbBr_2_ dissociation and complexation and energy costs required for surface adsorption from a selective solvent. Furthermore, we focus on surface adsorption at a low concentration of 1 mg/mL PS-*b*-PEO in *o*-xylene, at which micellization did not occur, and only free chains and clusters were present.

Munch et al. showed that the free energy of adsorption (*f*_ads_) of A-*b*-B block copolymers at a solid interface in a selective solvent consists of four contributions: (i) the elastic deformation energy of the copolymer chains in the adsorbed layer (*f*_def_), (ii) the mixing energy between solvent molecules and the outer-block chains (*f*_mix_), (iii) the interfacial energy per chain (*f*_int_), arising from repulsions between adsorbing blocks and solvent molecules, and (iv) the surface energy term (*f*_surf_), arising from attractions between adsorbing blocks and the solid surface.^62^

In addition, chain conformations of adsorbed polymer chains are also important. It is well established that physical adsorption of homopolymers or diBCPs onto a solid surface leads to three characteristic chain conformations: trains, loops, and tails.^65–70^ Trains correspond to segments directly adsorbed onto the surface, whereas loops are nonadsorbed segments connecting adjacent adsorption sites. These two conformations are typically associated with the adsorbing block. In contrast, nonadsorbing blocks extend into the good solvent and adopt tail-like conformations. The molecular weight of the adsorbing block is expected to play a key role in determining the distribution of trains and loops: long chains tend to form fewer trains but more loops, whereas short chains favor a higher density of trains and fewer loops.^71^ Large loops further induce a strong frustration in chain packing at an interfacial region.^72^ Notably, different from physical adsorption of polymers, surface-grafted polymers tend to exhibit brush- or mushroom-like conformations, which strongly depend on grafting density.^73,74^

Based on these considerations, we propose that the train–loop–tail framework and adsorption energetics reported in previous studies^62,65–72^ provides a plausible interpretation of the present observations. Specifically, PS_10k_-*b*-PEO_11.5k_ and PS_33k_-*b*-PEO_13.5k_ BCPs promote a high extent of PbBr_2_ dissociation and complexation, whereas PS_33k_-*b*-PEO_35k_, PS_38k_-*b*-PEO_102k_, and PS_140k_-*b*-PEO_141k_ BCPs lead to a much lower extent. For the former systems, short PEO blocks are proposed to favor adsorption conformations with a high density of trains and relatively few loops (Figure 6A). Such train-like conformations are expected to enhance direct contact between PEO ether groups and PbBr_2_ surfaces, which may facilitate PbBr_2_ dissociation and complexation. However, such adsorption also incurs both entropic penalties, arising from restricted chain mobility and chain deformation required to form train-rich conformations, and enthalpic penalties, associated with the exposure of PEO segments to *o*-xylene. Accordingly, *f*_def_ and *f*_int_ increase, respectively. The entropic penalty may be compensated by the surface energy term, *f*_surf_, which originates from attractive interactions between the adsorbing blocks and the solid surface. Meanwhile, the *f*_int_ penalty can be reduced when sufficiently long PS blocks effectively shield the adsorbed PEO chains from the solvent (Figure 6A).

**Figure 6:**
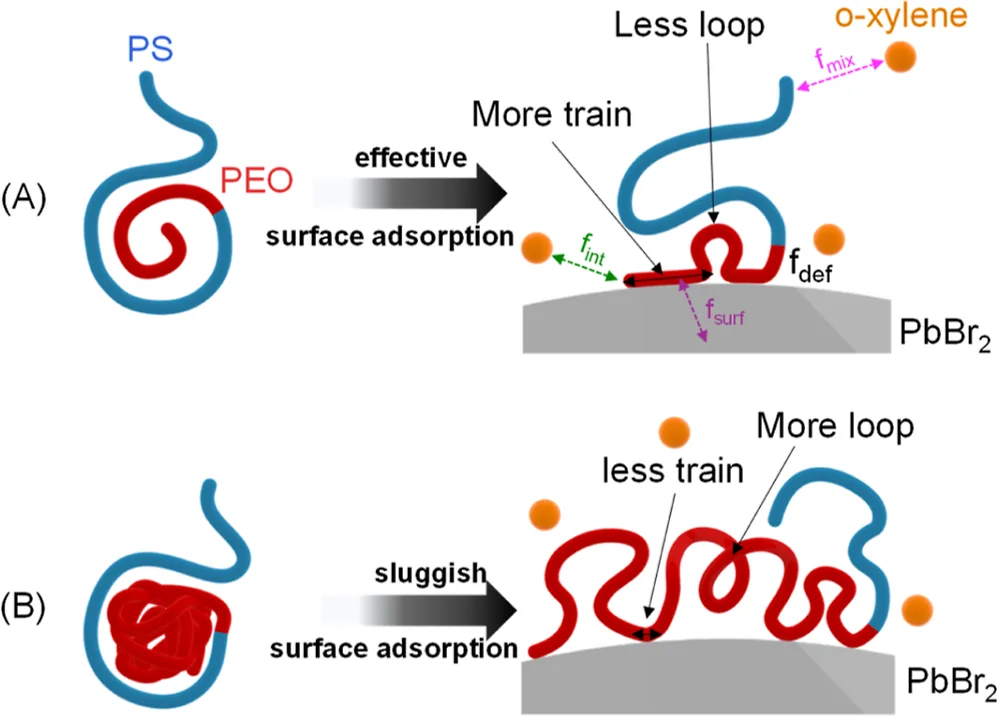
Proposed schematic illustration of two adsorption scenarios for PS-*b*-PEO chains with different block lengths on PbBr_2_ microparticle surfaces: (A) a diBCP with short PEO blocks and long PS blocks and (B) a diBCP with long PEO blocks and short PS blocks.

In contrast, for PS_33k_-*b*-PEO_35k_, PS_38k_-*b*-PEO_102k_, and PS_140k_-*b*-PEO_141k_, adsorption is expected to be dominated by conformations with a high density of loops and a low density of trains. In the case of PS_38k_-*b*-PEO_102k_, the PS block is too short to effectively screen the unfavorable interactions between PEO and *o*-xylene, resulting in a large interfacial energy penalty per chain (*f*_int_ ↑), which weakens adsorption onto PbBr_2_ surfaces (Figure 6B). For PS_33k_-*b*-PEO_35k_ and PS_140k_-*b*-PEO_141k_, although the PS and PEO blocks have comparable lengths, the predominance of loop conformations may introduce three adverse effects. First, the number of direct contacts between PEO ether groups and PbBr_2_ surfaces is expected to be limited. Because PbBr_2_ dissociation and complexation require close coordination between ether groups and Pb^2+^ ions, the reduced contact probability may significantly suppress these processes. Second, in the absence of sufficiently long PS chains, exposure of the PEO loop segments to *o*-xylene increases the enthalpic penalty associated with *f*_int_. To minimize this penalty, particle aggregation becomes more favorable. Third, loop conformations are expected to increase interparticle bridging, as reported by Cheng et al.^72^ These factors may collectively explain the poor particle dispersion observed in t-PRE_33/35_, t-PRE_38/102_, and t-PRE_140/141_ solutions.

Although the train–loop–tail framework provides a useful basis for interpreting the present observations, quantitative determination of the fractions of train, loop, and tail conformations remains highly challenging for physically adsorbed block copolymers, particularly on irregular microparticles with broad size distributions. Unlike surface-grafted polymer brushes, physically adsorbed chains may undergo dynamic adsorption–desorption and continuous conformational rearrangements driven by Brownian motion and particle collisions in solution, making direct experimental quantification difficult. Moreover, in the present system, the structural heterogeneity and broad size distribution of the PbBr_2_ microparticles, together with the interfacial PbBr_2_ dissociation and complexation processes, further complicate quantitative analysis. A recent review of polymer–particle interfaces has further emphasized that quantitative characterization of adsorbed polymer conformations generally requires the integration of multiple complementary experimental techniques with theoretical or computational approaches, rather than a single characterization method.^75^ Development of reliable experimental and theoretical approaches for quantitatively resolving train–loop–tail conformations in physically adsorbed block copolymers therefore remains an important topic for future investigation.

## Conclusions

We have demonstrated that the volume fraction and molecular weight of PS-*b*-PEO block copolymers play critical roles in governing the dispersion, dissociation, and complexation of PbBr_2_ microparticles in *o*-xylene at the polymer concentration investigated (1 mg/mL), where well-defined micelles were not detected. Asymmetric PS-*b*-PEO diblock copolymers with long PS blocks and short PEO blocks promote more efficient dispersion, dissociation, and complexation of PbBr_2_ microparticles during stirring, whereas copolymers with short PS blocks and long PEO blocks exhibit substantially lower efficiencies. For symmetric diblock copolymers with comparable block volume fractions, molecular weight also plays an important role, with lower-molecular-weight PS-*b*-PEO exhibiting higher degrees of PbBr_2_ dispersion, dissociation, and complexation than their higher-molecular-weight counterparts.

On the basis of the experimental observations together with previous studies of block copolymer adsorption, we propose that these differences originate from two factors. First, long PS blocks are expected to better screen unfavorable interactions between PEO segments (or adsorbed PEO chains) and *o*-xylene, thereby stabilizing dispersed PbBr_2_ particles and the resulting hybrid mesostructures. Second, short PEO blocks are proposed to favor adsorption conformations containing a higher density of train segments and fewer loops, leading to more effective interfacial contact with PbBr_2_ surfaces and, consequently, facilitating PbBr_2_ dissociation and complexation. In contrast, long PEO blocks are proposed to form fewer train segments and more loops, thereby reducing the effective interfacial contact.

## Supplementary Material



## Notes

SAXS profiles of diBCP_*m*/*n*_ solutions plotted as absolute intensity as a function of *q*; structural parameters used to fit the SAXS profiles; fitting residuals; AFM topographic images; theoretical calculations of unperturbed PS-*b*-PEO diBCP chains; stability and Tyndall-effect tests of aged t-PRE_*m*/*n*_ solutions; SAXS profiles of t-PRE_*m*/*n*_ solutions plotted as absolute intensity as a function of *q*; structural parameters used to fit the SAXS profiles; fitting residuals; in-house WAXD profiles of control samples; LV-TEM, ED, and EDS data; and TEM images of dried t-PRE_m/n_ samples prepared from 24 h-aged t-PRE_*m*/*n*_ solutions (PDF)
