# Supplementary material for: Effect of Block Copolymer Architecture on Lead(II) Bromide (PbBr2) Dissociation and Complexation via Surface Adsorption in a Selective Solvent
Source: Langmuir. 2026 Aug 27;42(36):26290–302. doi: 10.1021/acs.langmuir.6c02844 (PMC13585135; doi:10.1021/acs.langmuir.6c02844)
Supplement: Supplementary file 1 [file la6c02844_si_001.pdf]

Supporting Information

Effect of Block Copolymer Architecture on Lead(II) Bromide ($PbBr_2$) Dissociation and Complexation via Surface Adsorption in a Selective Solvent.

Ya-Sen Sun* and Yu-Chieh Chen

Department of Chemical Engineering, National Cheng Kung University, Tainan 701, Taiwan

*corresponding author: Y. S. Sun (Email: yssun@gs.ncku.edu.tw)

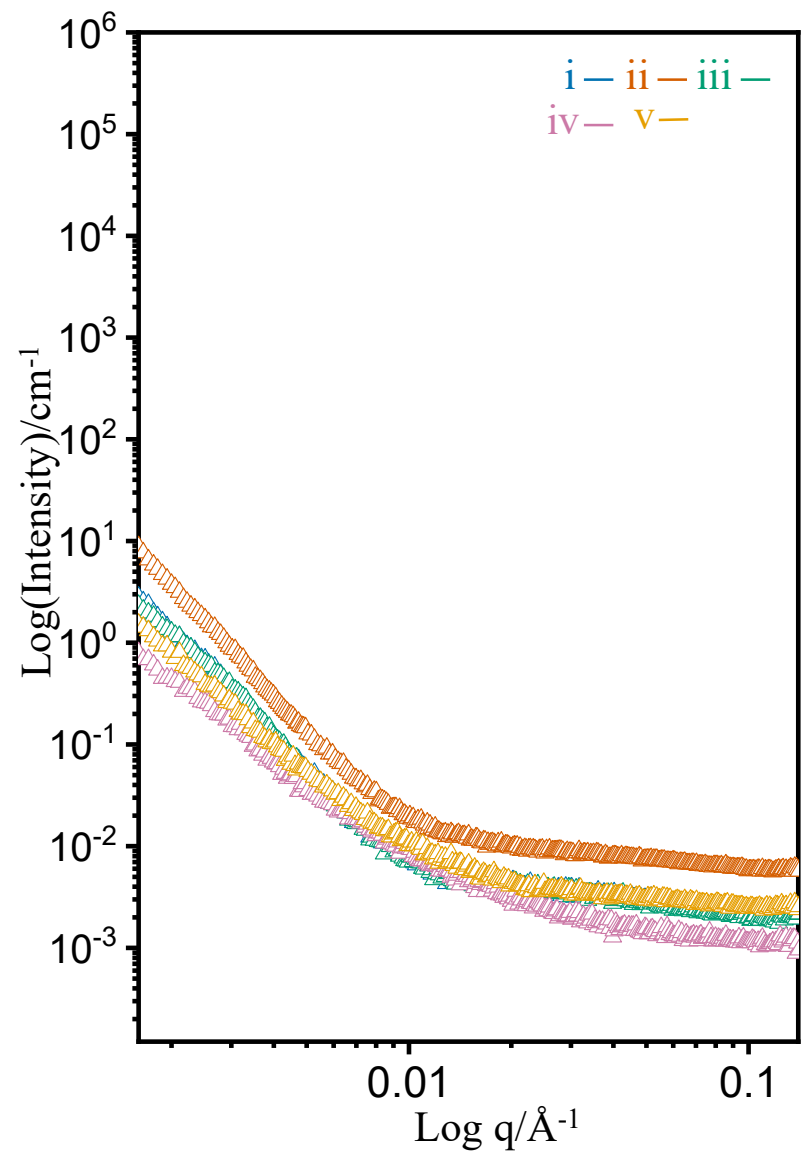

Figure S1. SAXS profiles, presented in absolute intensity as a function of *q*, of $diBCP_{m/n}$ solutions with m/n = (i)10/11.5, (ii)33/13.5, (iii) 33/35, (iv) 38/102, and (v) 140/141.

Table S1. Structural Parameters used to fit Figure 1C.

PS_{mk}-b-PEO_{nk} with m/n =	$R_{g,s}$ (Å)	α_s	$R_{g,l}$ (Å)	α_l	χ^2
	Two-level Beaucage model				
**10/11.5**	36.2 ± 1.30	0.35	1871.5 ± 126	3.40	1.68
**33/13.5**	46.7 ± 0.74	0.32	1985.4 ± 109	3.75	2.05
**33/35**	44.3 ± 0.95	0.76	1502.9 ± 57	3.98	1.37
**38/102**	51.4 ± 0.9	0.64	2371 ± 101	2.65	1.81
**140/141**	63.6 ± 2.20	0.3	1163 ± 21	3.0	1.20

Note: The subscripts s and l denote "small" and "large", respectively.

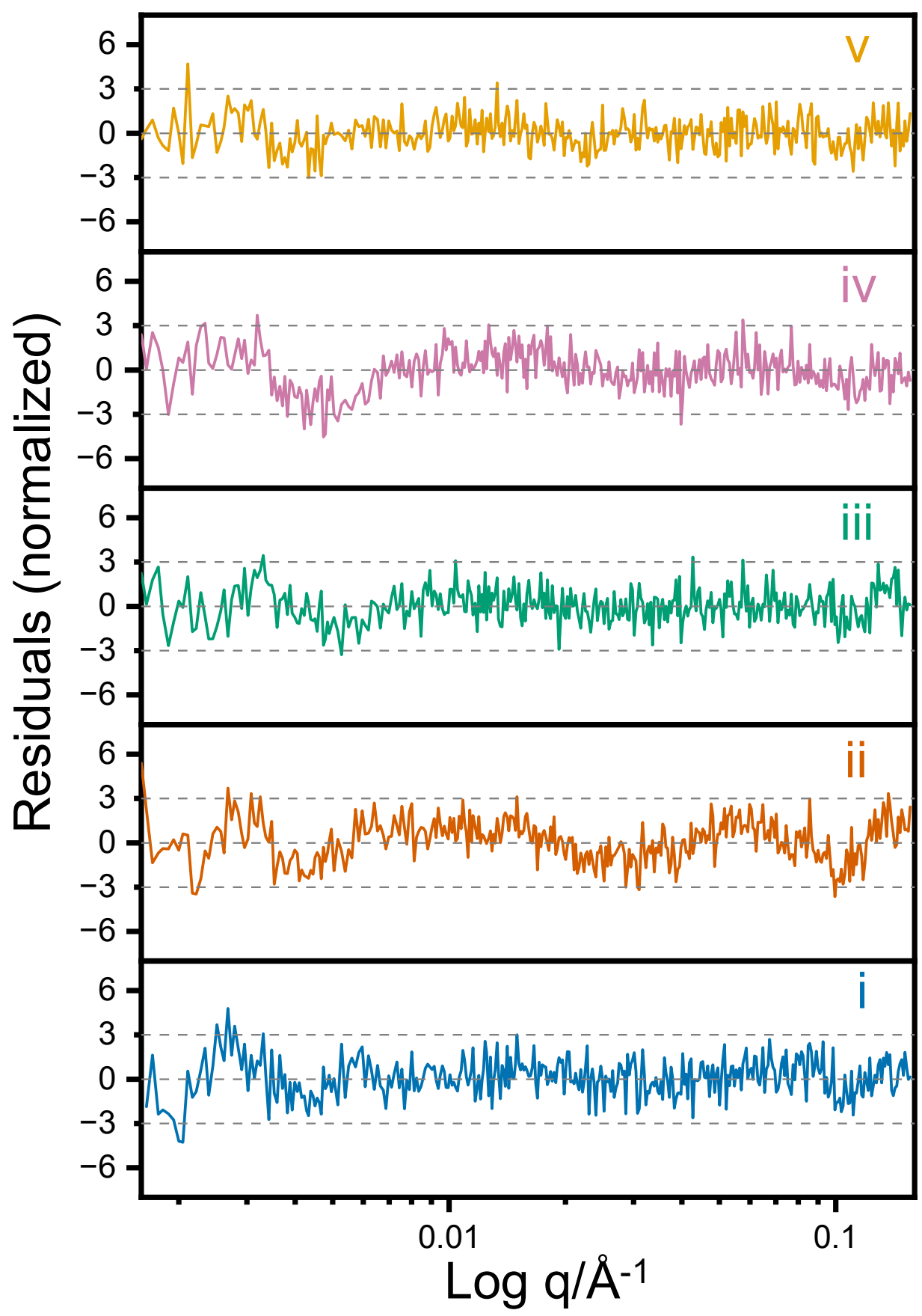

Figure S2. Fitting residuals of Figure 1C.

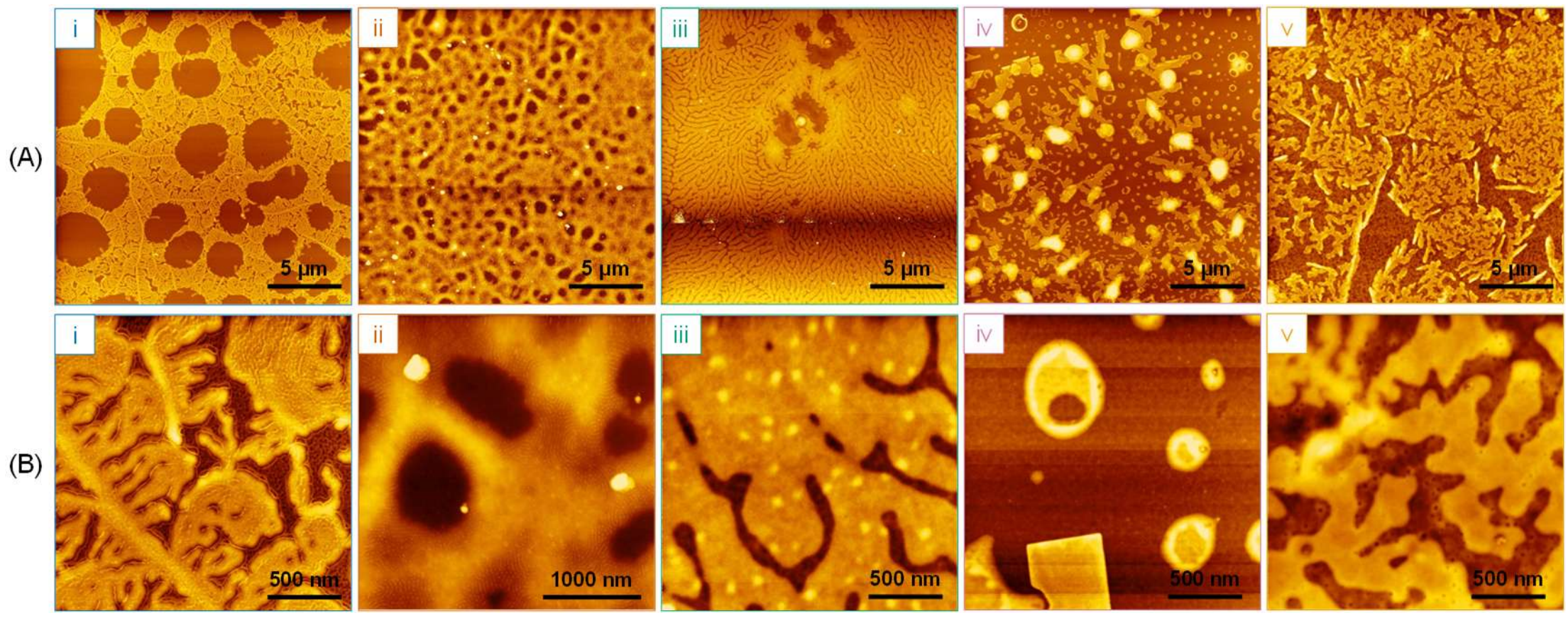

Figure S3. AFM topographic images of $diBCP_{m/n}$ dried samples with m/n = (i) 10/11.5, (ii) 33/13.5, (iii) 33/35, (iv) 38/102, and (v) 140/141.

Calculation of the radius of gyration of unperturbed PS-b-PEO chains

According to previous studies[S1-S2], we theoretically calculated the radius of gyration (R_g^o) of an unperturbed PS-*b*-PEO chain, given by

$$R_g^o = \frac{a_{PS/PEO}}{6^{1/2}} N^{1/2} \qquad \text{(S1)}$$

with $$a_{PS/PEO} = (\frac{f_{PS}}{a_{PS}^2} + \frac{f_{PEO}}{a_{PEO}^2})^{-1/2} \qquad \text{(S2)}$$

In equations (S1) and (S2), a_i denotes the segmental length of constituent block i, and f_i is the volume fraction of constituent block i. For the R_g^o values summarized in Table 1, a_{PS}=0.67 nm and a_{PEO}=0.6 nm, and ρ_{PS}=1.05 g/mL and ρ_{PEO}=1.13 g/mL were used to the calculation.[S3-S4]

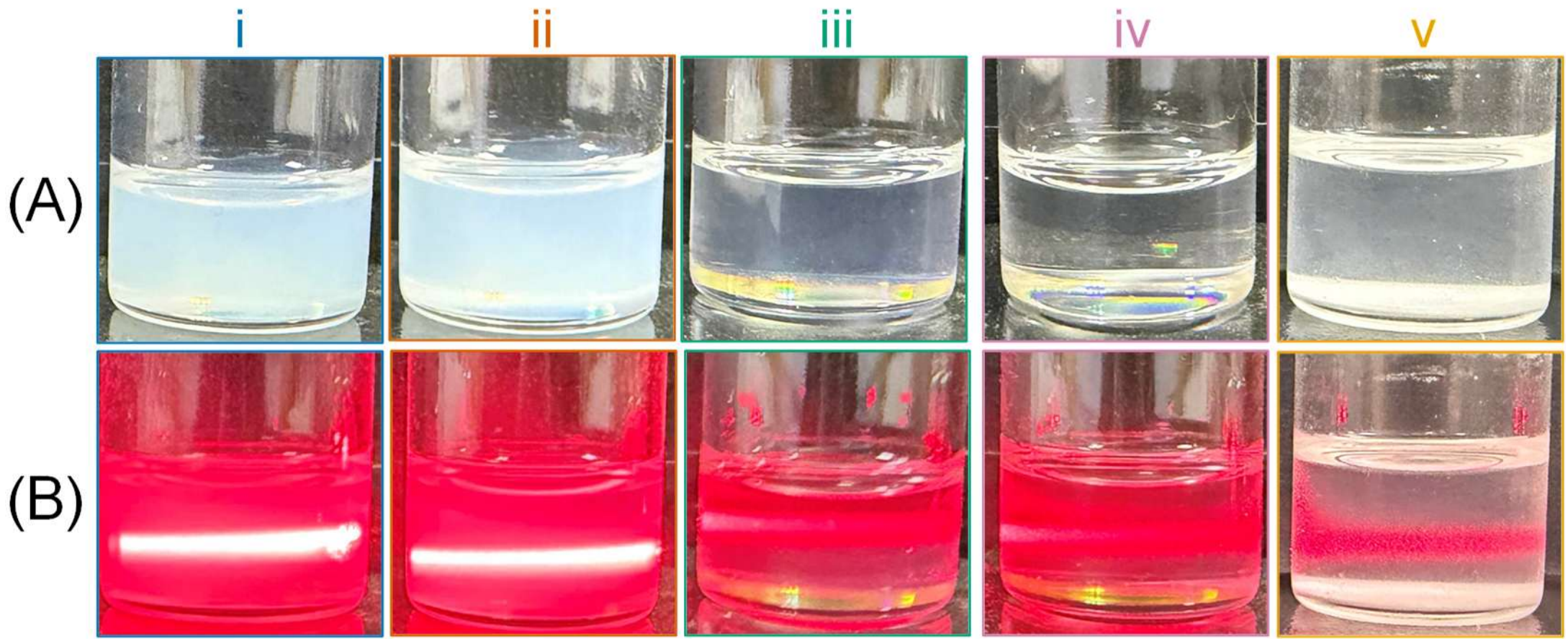

Figure S4. (A) Photos and (B) Tyndall tests of t-PRE$_{m/n}$ solutions with m/n = (i)10/11.5, (ii)33/13.5, (iii) 33/35, (iv) 38/102, and (v) 140/141 after 24 h of static aging at room temperature following the cessation of stirring.

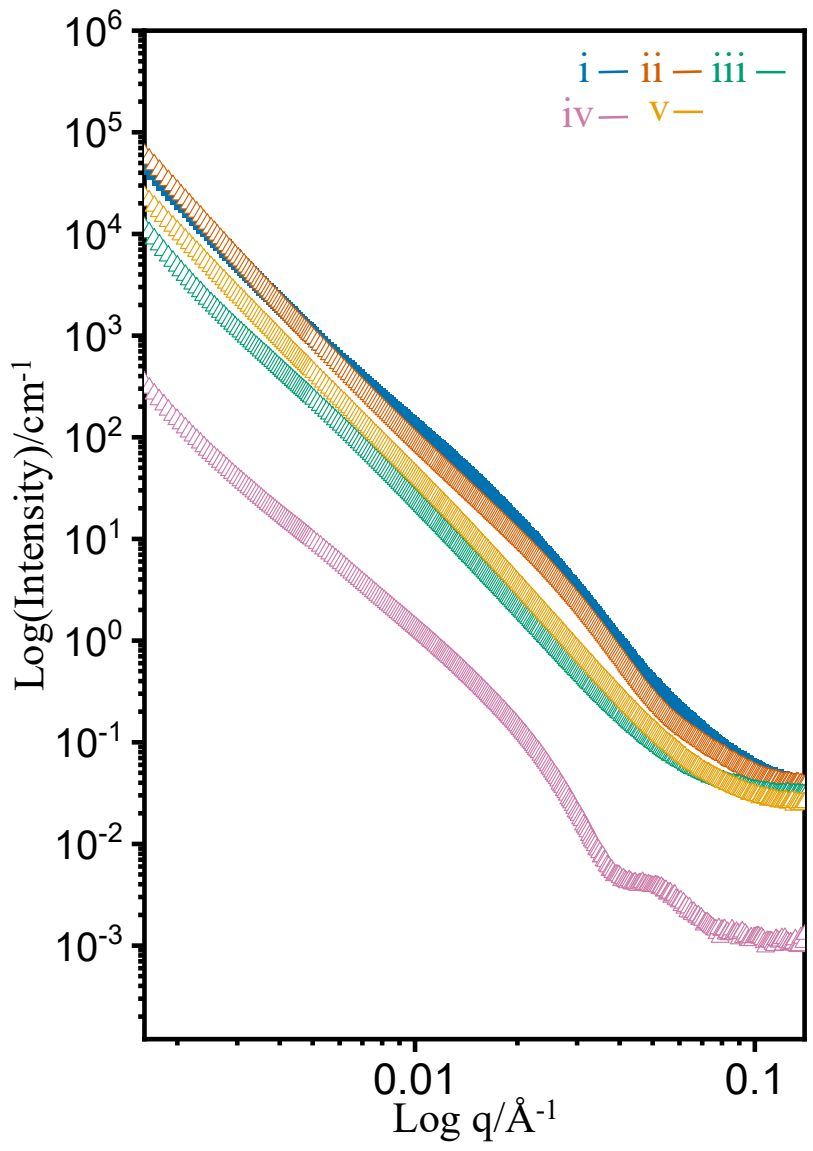

Figure S5. SAXS profiles, presented in absolute intensity as a function of *q*, of t-$PRE_{m/n}$ solutions with m/n = (i)10/11.5, (ii)33/13.5, (iii) 33/35, (iv) 38/102, and (v) 140/141.

Table S2. Structural Parameters used to fit Figure 4D.

t-$PRE_{m/n}$ with m/n =	$R_{g,s}$ (Å)	α_s	$R_{g,l}$ (Å)	α_l	χ^2
	Two-level Beaucage model				
**10/11.5**	100 ± 0.12	1.84	703 ± 0.50	3.27	80
**33/13.5**	70.3 ± 0.10	1.51	667.7 ± 0.65	3.24	66
**33/35**	86 ± 0.02	0.75	1412 ± 2.60	3.30	17.6
**38/102**	110 ± 0.10	1.32	1562 ± 1.10	3.98	9.6
**140/141**	155 ± 0.56	1.5	892.1 ± 4.0	3.32	5.5

Note: The subscripts s and l denote "small" and "large", respectively.

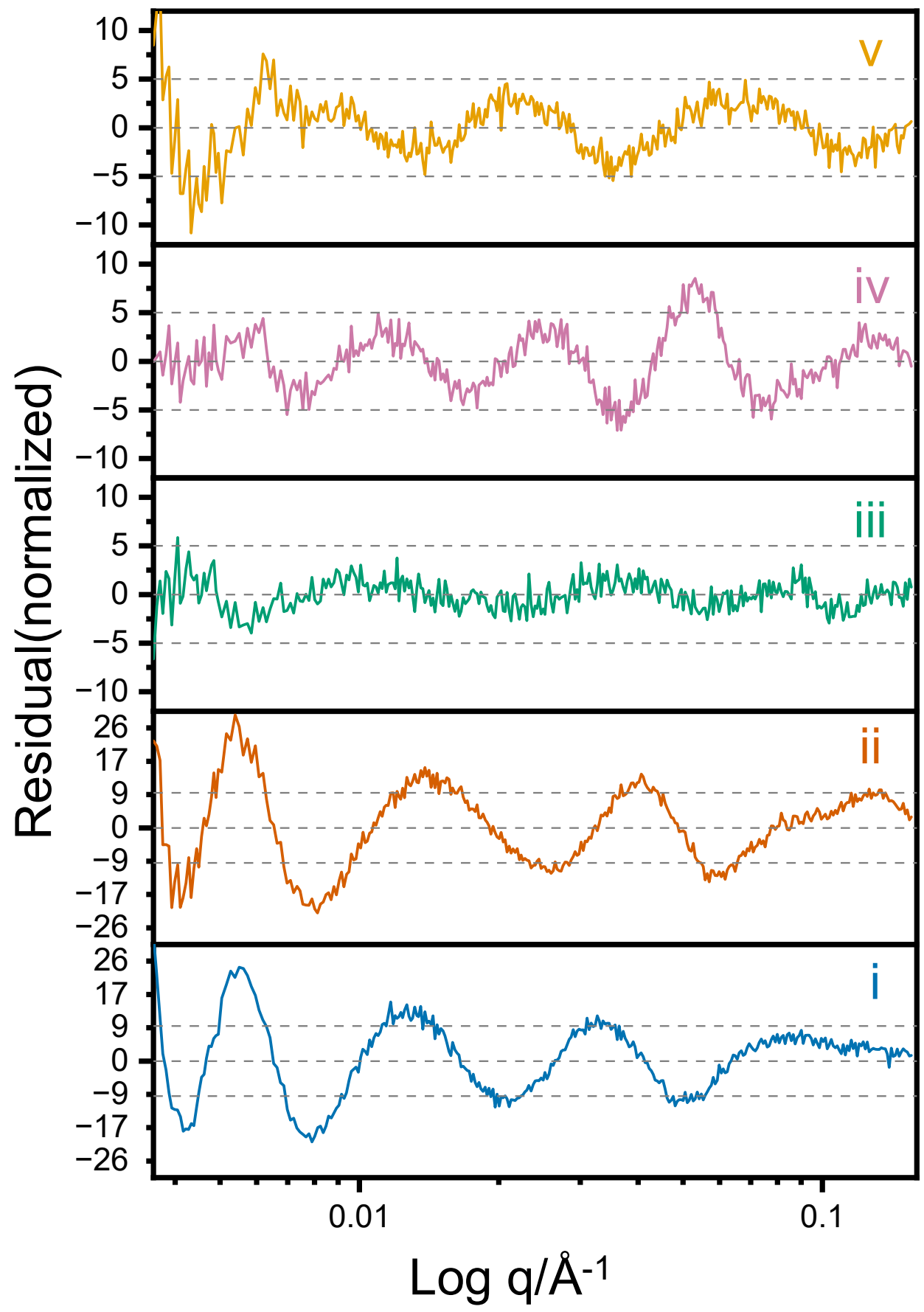

Figure S6. Fitting residuals of Figure 4D.

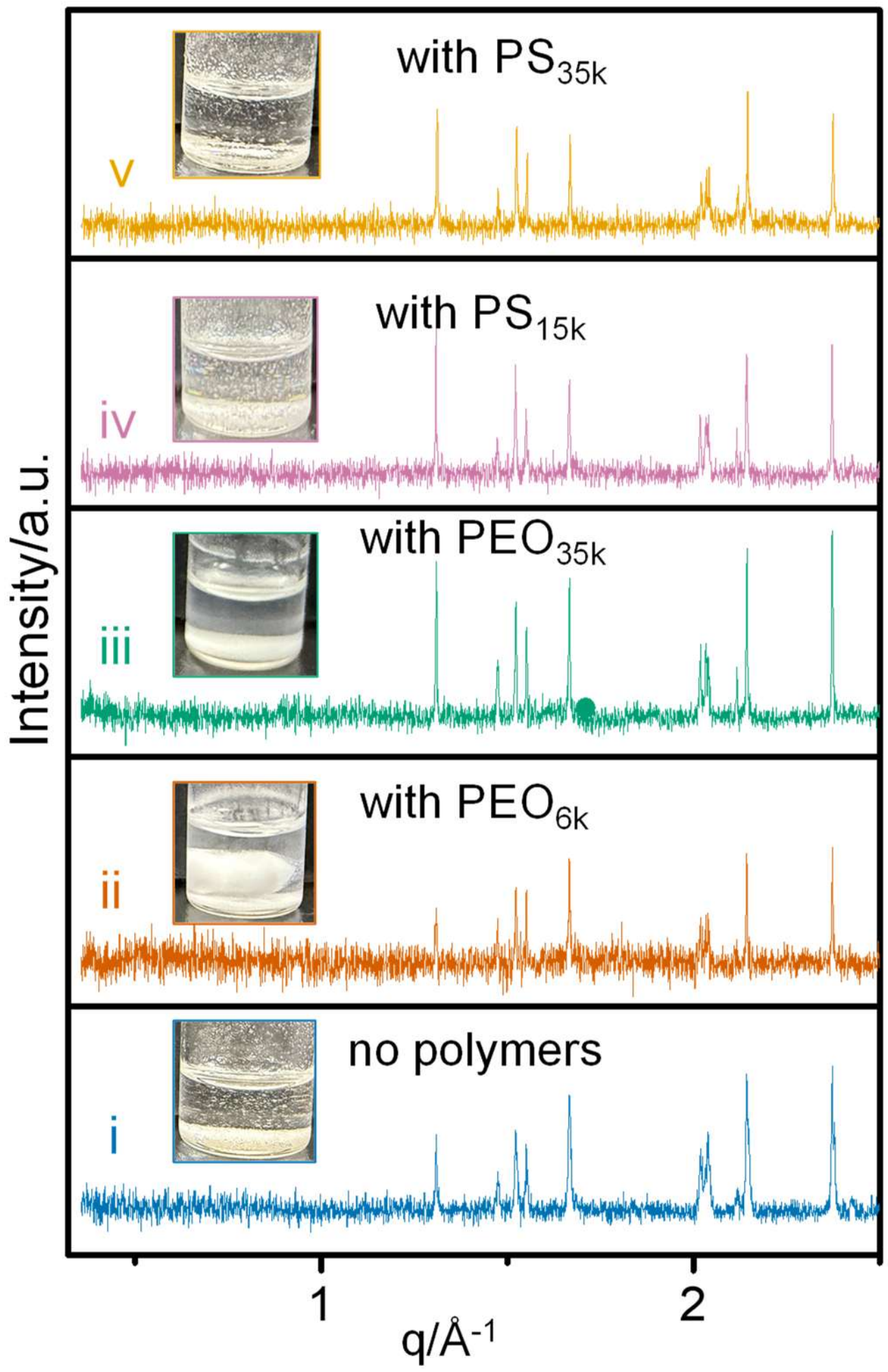

Figure S7. In-house WAXD profiles and photographs of five control samples. The control samples were prepared by dispersing 10 mg of $PbBr_2$ in (i) polymer-free *o*-xylene, (ii, iii) *o*-xylene containing 1 mg mL^{-1} PEO (PEO$_{6k}$ and PEO$_{35k}$), and (iv, v) *o*-xylene containing 1 mg mL^{-1} PS (PS$_{15k}$ and PS$_{35k}$). All control samples were stirred at 800 rpm for 48 h under the same conditions used for the PS-*b*-PEO systems. Because of the poor dispersion of $PbBr_2$ in these control solutions, the $PbBr_2$ microparticles remained poorly dispersed (see the insets). After 48 h of stirring, the $PbBr_2$ precipitates were collected for in-house WAXD characterization.

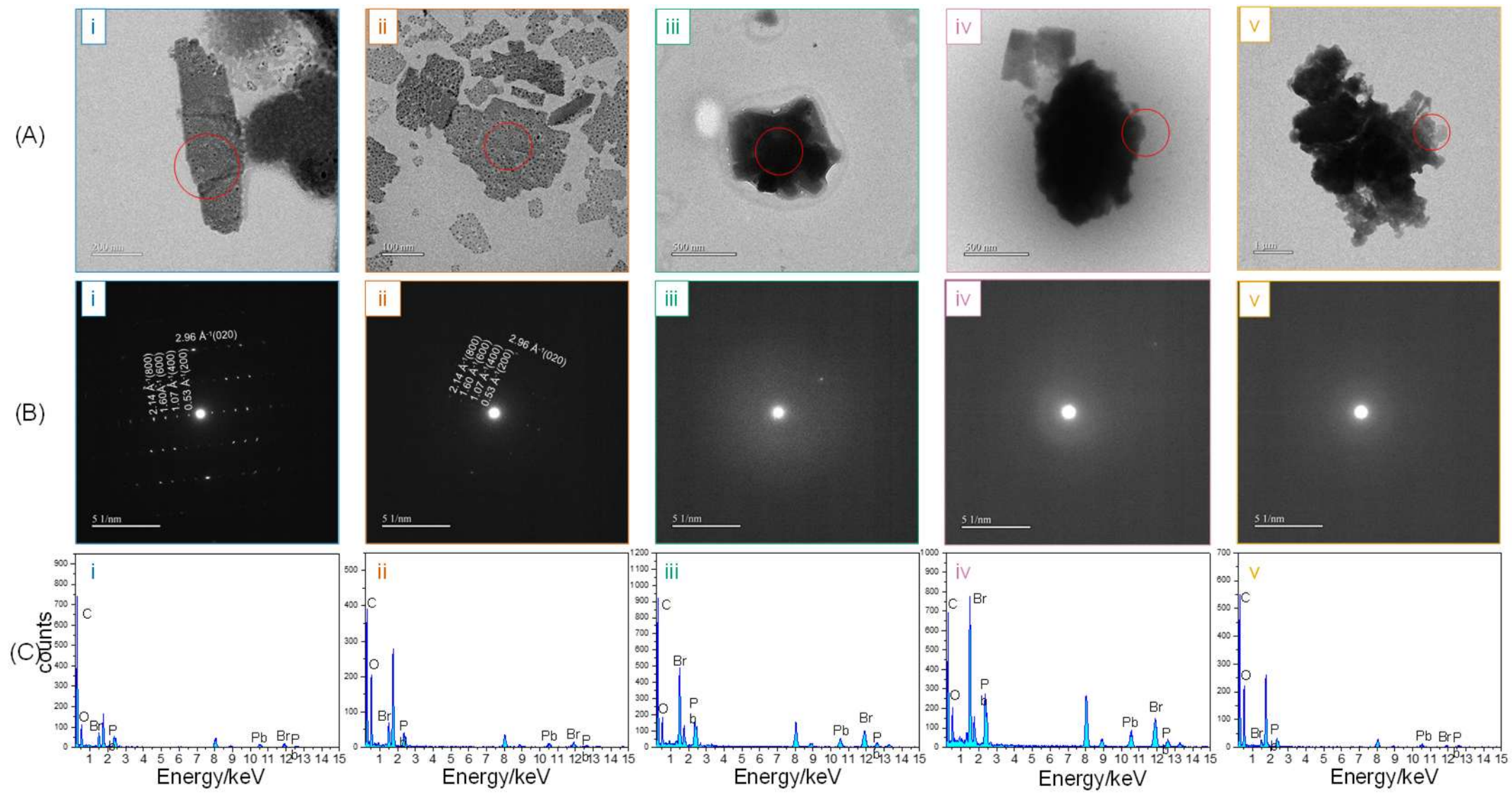

Figure S8. (A) LV-TEM images, (B) ED patterns, and (C) EDS spectra of dried t-$PRE_{m/n}$ samples prepared from t-$PRE_{m/n}$ solutions with m/n = (i)10/11.5, (ii)33/13.5, (iii) 33/35, (iv) 38/102, and (v) 140/141. The regions selected for ED and EDS analyses are indicated by the red circles in (A).

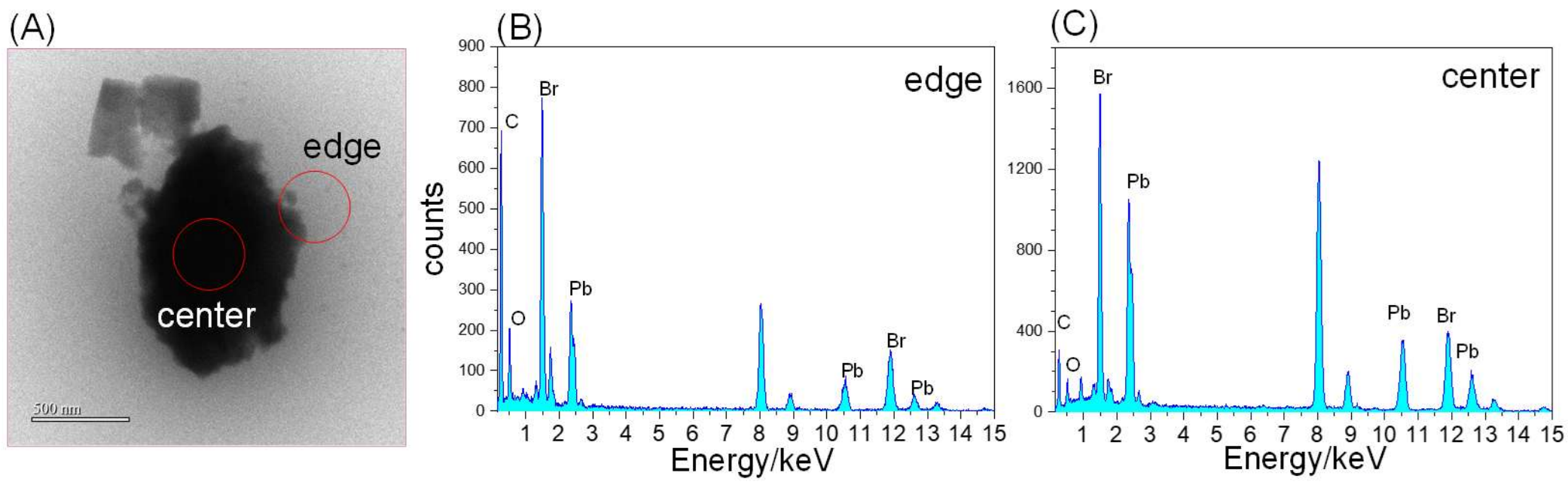

Figure S9. (A) LV-TEM image and (B, C) EDS spectra acquired from the edge and center regions, respectively, of a representative $PbBr_2$ microparticle formed in the t-$PRE_{38/102}$ solution. The EDS acquisition regions are indicated by the red circles in (A).

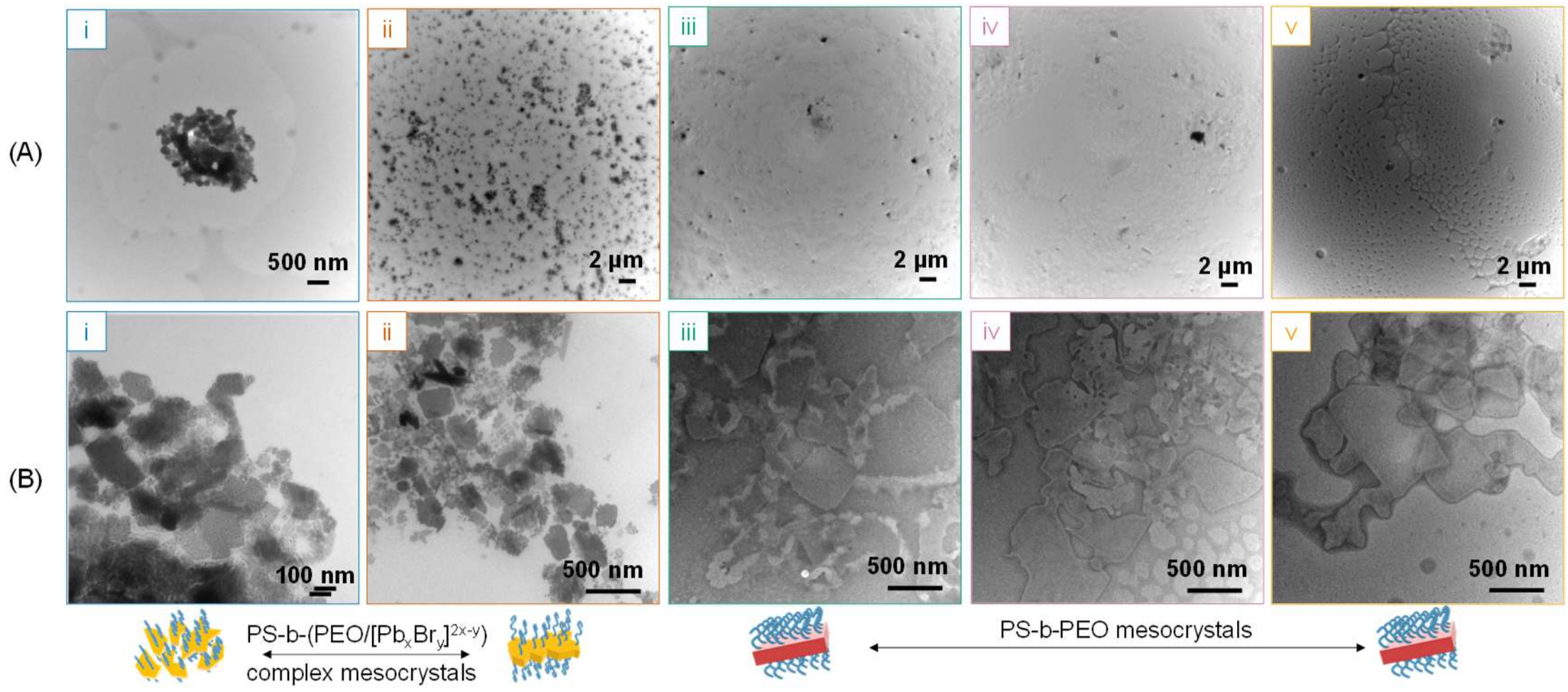

Figure S10. (A) Low- and (B) high-magnification TEM images of dried t-PRE$_{m/n}$ samples prepared from t-PRE$_{m/n}$ solutions after 24 h of static aging at room temperature with m/n = (i)10/11.5, (ii)33/13.5, (iii) 33/35, (iv) 38/102 and (v)140/141.

References:

[S1] Sun, Y. S.; Chung, T. M.; Li, Y. J.; Ho, R. M.; Ko, B. T.; Jeng, U. S.; Lotz, B. Crystalline Polymers in Nanoscale 1D Spatial Confinement. *Macromolecules* **2006**, *39*, 5782-5788.

[S2] Septani, C. M.; Ku, M.-F.; Chen, C.-Y.; Lin, J.-M.; Sun, Y.-S. Micellization, Aggregation, and Gelation of Polystyrene-*block*-poly(ethylene oxide) in Cosolvents Added with Hydrochloric Acid. *Colloids Surf. A: Physicochem. Eng. Asp*. **2022**, *652*, 129857.

[S3] Hiemenz, P. C.; Lodge, T. P. Polymer Conformation. In *Polymer Chemistry*, 2nd ed.; CRC Press, Taylor & Francis Group: Boca Raton, FL, 2007; Chapter 6, pp 217-242.

[S4] Fetters, L. J.; Lohse, D. J.; Richter, D.; Witten, T. A.; Zirkel, A. Connection between Polymer Molecular Weight, Density, Chain Dimensions, and Melt Viscoelastic Properties. *Macromolecules* **1994**, *27*, 4639-4647.
